# Hyperspectral unmixing for Raman spectroscopy via physics-constrained autoencoders

**DOI:** 10.1073/pnas.2407439121

**Published:** 2024-10-29

**Authors:** Dimitar Georgiev, Álvaro Fernández-Galiana, Simon Vilms Pedersen, Georgios Papadopoulos, Ruoxiao Xie, Molly M. Stevens, Mauricio Barahona

**Affiliations:** ^a^Department of Computing, Faculty of Engineering, Imperial College London, London SW7 2AZ, United Kingdom; ^b^UKRI Centre for Doctoral Training in AI for Healthcare, Imperial College London, London SW7 2AZ, United Kingdom; ^c^Department of Materials, Faculty of Engineering, Imperial College London, London SW7 2AZ, United Kingdom; ^d^Department of Bioengineering, Faculty of Engineering, Imperial College London, London SW7 2AZ, United Kingdom; ^e^Institute of Biomedical Engineering, Faculty of Engineering, Imperial College London, London SW7 2AZ, United Kingdom; ^f^Medical Sciences Division, Department of Physiology, Anatomy and Genetics, University of Oxford, Oxford OX1 3QU, United Kingdom; ^g^Mathematical, Physical & Life Sciences Division, Department of Engineering Science, University of Oxford, Oxford OX1 3QU, United Kingdom; ^h^Medical Sciences Division and Mathematical, Physical & Life Sciences Division, Kavli Institute for Nanoscience Discovery, University of Oxford, Oxford OX1 3QU, United Kingdom; ^i^Department of Mathematics, Faculty of Natural Sciences, Imperial College London, London SW7 2AZ, United Kingdom

**Keywords:** Raman spectroscopy, hyperspectral unmixing, machine learning, autoencoders, chemometrics

## Abstract

Raman spectroscopy is widely used across scientific domains to characterize the chemical composition of samples in a nondestructive, label-free manner. Many applications entail the unmixing of signals from mixtures of molecular species to identify the individual components present and their proportions, yet conventional methods for chemometrics often struggle with complex mixture scenarios encountered in practice. Here, we develop hyperspectral unmixing algorithms based on autoencoder neural networks, and we systematically validate them using both synthetic and experimental benchmark datasets created in-house. Our results demonstrate that unmixing autoencoders provide improved accuracy, robustness, and efficiency compared to standard unmixing methods. We also showcase the applicability of autoencoders to complex biological settings by showing improved biochemical characterization of volumetric Raman imaging data from a monocytic cell.

## Significance

Hyperspectral unmixing methods are essential to exploit the capabilities of Raman spectroscopy for nondestructive, unbiased chemical characterization in a wide array of domains, from biology, chemistry, and materials to engineering and environmental science. Here, we take advantage of recent advances in machine learning and introduce a framework for Raman unmixing based on autoencoder neural networks. We demonstrate that such methods offer more versatile, robust, and data-driven Raman unmixing with improved performance compared to conventional methods in complex samples.

Raman spectroscopy (RS) is a powerful optical modality that facilitates the identification, characterization, and quantification of the molecular composition of chemical and biological specimens, offering in-depth insights into their structure and functionality (1–5). RS interrogates the vibrational modes of molecules through the analysis of inelastic scattering of monochromatic light from matter, thereby enabling the nondestructive, label-free fingerprinting of chemical species (6–10). As a result, RS has become an important analytical tool in a myriad of scientific domains, from chemistry (11, 12), biology (13–16), and medicine (17–23), to materials science (24, 25), pharmacology (26–28), environmental science (29–31), food quality control (32, 33), and even forensics (34–36).

Despite the wealth of information RS affords, the analysis and interpretation of experimental RS data remains a major challenge (37–39). Many important applications entail the analysis of complex mixtures of molecular species coexisting and interacting at micro- and nanoscales. Such complexity can hinder the qualitative and quantitative investigation of RS measurements, especially when dealing with the biomolecular diversity of biological samples (39, 40).

**Fig. 1.**
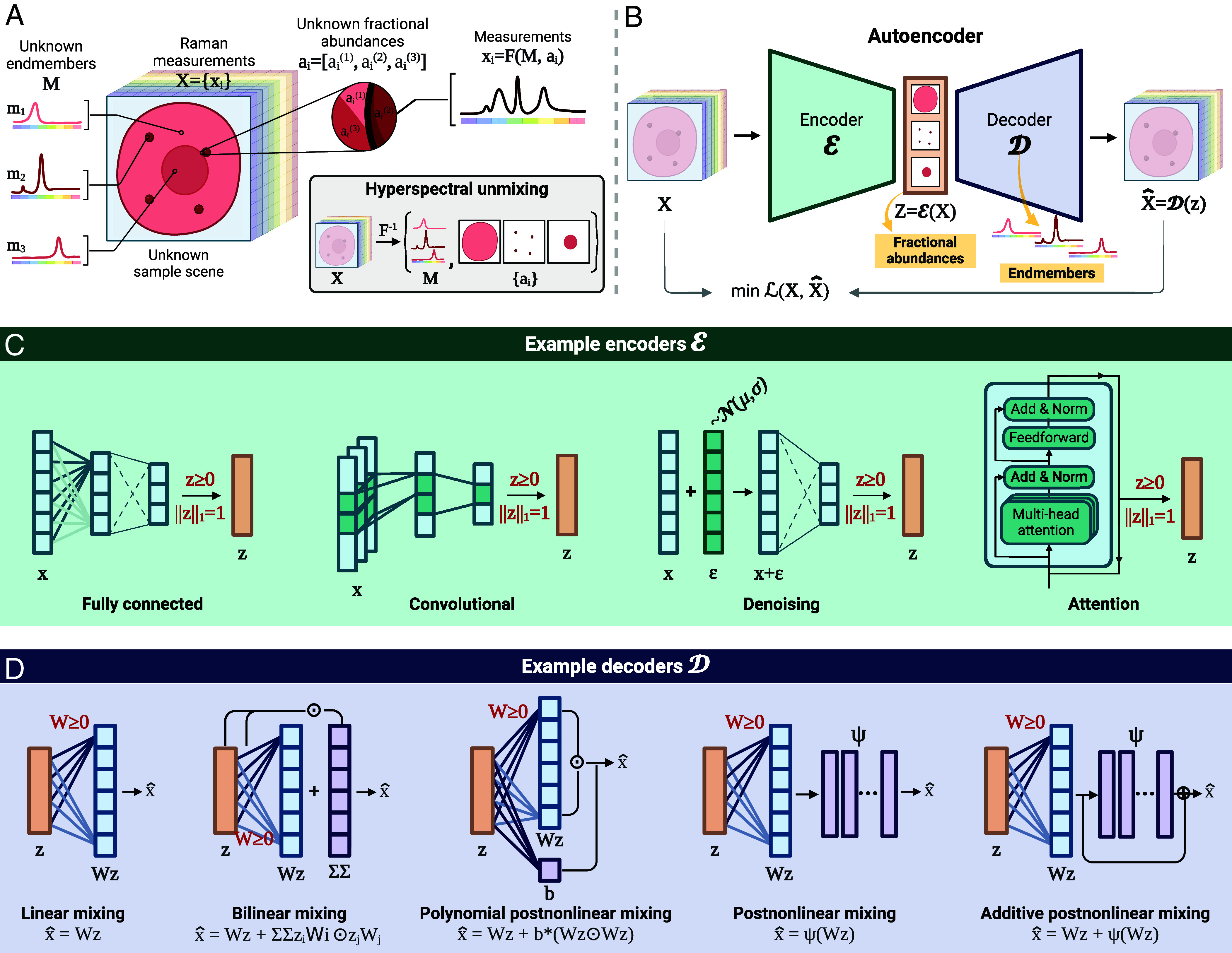
Hyperspectral unmixing for Raman spectroscopy using autoencoder neural networks. (*A*) Illustration of hyperspectral unmixing. A set of Raman measurements $X=\{x_{i}\}$, where each $x_{i}$ is a spectrum, are collected from a sample of interest. The spectra $x_{i}$ are viewed as mixtures of a set of Raman spectral signatures M (endmembers), related to the chemical species present, and their respective fractional abundances $a_{i}$. Hyperspectral unmixing aims to infer the set of unknown underlying endmembers M and fractional abundances $a_{i}$ from the set of measurements X, thereby obtaining qualitative and quantitative compositional information about the sample. (*B*) Hyperspectral unmixing as a self-supervised autoencoder learning problem: the decoder D learns endmember signatures during training and the encoder E learns to derive the corresponding fractional abundances. (*C*) Encoders can accommodate different concepts from representation learning, such as convolutional layers and attention, to improve feature extraction and provide more accurate and robust unmixing. (*D*) Decoders can be structured to model different linear and nonlinear mixing models. Physics-inspired constraints are indicated in red in *C* and *D*.

Hyperspectral unmixing (also known as (hyper)spectral deconvolution or multivariate curve resolution) aims to resolve such mixed signals (41, 42) by identifying the individual components present (endmember identification) and/or quantifying their proportions (abundance estimation) (Fig. 1*A*). Popular approaches include N-FINDR (43) and vertex component analysis (VCA) (44) for endmember identification, and non-negative least squares (NNLS) (45) and fully constrained least squares (FCLS) (46) for abundance estimation (41, 47). However, such techniques, which originated in the field of remote sensing (48, 49), have limitations for the unmixing of RS data. Specifically, these methods are typically restricted to linear mixing; lack robustness to data artifacts abundant in RS data (e.g., dark noise, baseline variations, cosmic spikes); rely on additional assumptions (e.g., endmembers present as “pure pixels” in the data) or require additional information (e.g., number of endmembers, underlying mixture model, endmember library); and are computationally demanding for large datasets (e.g., imaging and volumetric Raman raster scans).

Over the past few years, machine learning (ML) has gained considerable traction for the analysis of Raman spectroscopy data (50–54). Nonetheless, despite significant advances, most methods remain focused on constrained supervised tasks where a model is learned assuming knowledge of target species and their values (55). In particular, approaches for compositional characterization of Raman measurements normally utilize explicit information provided during model training about the presence (or absence) of particular target species (56) or known concentrations (57–61). Therefore, the development of methods for general unsupervised hyperspectral unmixing that do not rely on known target substances and concentrations remains essential to realize the potential of Raman spectroscopy for label-free chemical characterization.

Autoencoder (AE) neural networks have recently emerged as a framework for hyperspectral unmixing in remote sensing, spurred by the availability of standardized benchmark datasets for model evaluation (e.g., *Urban*, *Samson*, *AVARIS Cuprite*) (62–66). Yet, despite initial explorations (67, 68), the utility of unmixing AEs for Raman spectroscopy data remains largely unexplored. Here, we develop a range of AEs for RS hyperspectral unmixing, which we systematically validate against conventional unmixing methods using synthetic and experimental Raman data.

## Results

### Raman Unmixing with Autoencoder Neural Networks.

Autoencoders are a family of (deep) neural network models consisting of two subnetworks (encoder and decoder) connected sequentially (69). The encoder $E:R^{b}\to R^{m}$, where m≪b, transforms input data x to a lower-dimensional latent space representation z=E(x), which the decoder $D:R^{m}\to R^{b}$ uses to produce reconstructions $\hat{x}=D\left(z\right)$ of the original input. AE models are typically trained in a self-supervised manner by minimizing a loss function $L(x,\hat{x})$ that measures the discrepancy between the input x and the reconstruction $\hat{x}$ (e.g., the mean squared error (MSE)); hence no ground-truth target values are provided during training. As the training of the model proceeds, the encoder progressively learns a latent representation that captures the most salient features of the input data, whereas the decoder learns how to recover the data back from the latent representation.

This dual functionality can be harnessed to design autoencoders for hyperspectral unmixing: the latent representations z=E(x) can be interpreted as fractional abundances (with respect to the input spectrum x), and the decoder D(·) acts as a mixing function on these representations by encoding endmember signatures and other interactions. Hence, AE models learn to perform “unmixing” where the decoder identifies endmember signatures and the encoder quantifies the fractional abundances of these learned endmembers in the input spectrum (Fig. 1*B*). To guide the learning, we incorporate physical constraints into the AE architecture to reflect the nature of hyperspectral unmixing, e.g., nonnegativity of endmembers and fractional abundances, and sum-to-one abundances (Fig. 1 and *Materials and Methods*).

Compared to standard methods for unmixing, autoencoders are more effective at capturing complex, noisy, and nonlinear relationships in the data, and do not rely on assumptions like the presence of pure pixels (see *SI Appendix*, Table S1, for extended discussions). Moreover, AEs offer a more flexible and versatile framework for unmixing, as illustrated by the wide design space of encoder and decoder architectures (Fig. 1 *C* and *D*).

On the one hand, the learning of physical and biochemical features in the encoder can be enhanced by adopting strategies from representation learning, such as convolutional layers to capture spectral and/or spatial correlations among neighboring bands and/or pixels (70–72), or attention mechanisms to model long-range dependencies (73) (Fig. 1*C*). In addition, sparsity, part-based learning, and denoising objectives can be enforced during training to enhance explainability and robustness (74–78).

On the other hand, the design of the decoder allows for flexible modeling of input data, specifically to account for various mixture models, e.g., linear, bilinear, and post-nonlinear (Fig. 1*D*) (66, 79, 80). This is akin to introducing an inductive prior with respect to the mixture model directly via the AE architecture. Furthermore, the decoder can be preinitialized with a set of endmembers (e.g., an endmember library or signatures derived using methods such as VCA), or readily adapted to nonblind unmixing by fixing certain parameters to predefined endmember signatures.

#### Unmixing autoencoders.

To assess the effectiveness of AEs as a framework for RS unmixing, we develop and evaluate a collection of AE models, each defined by a specific encoder and decoder. We consider four types of encoders encompassing a variety of architectures, from traditional dense layers to contemporary convolutional and attention mechanisms: 1) an encoder consisting of fully connected layers (*Dense*); 2) an encoder with a 1D convolutional feature extractor block, followed by a fully connected part (*Convolutional*); 3) a transformer-based encoder that uses multi-head attention (*Transformer*) (81); and 4) a transformer-based encoder with a 1D convolutional feature extractor (*Convolutional Transformer*). The two types of decoders we investigate are 1) a decoder designed for linear unmixing (Eq. **4**) and 2) a decoder designed for bilinear unmixing (Eq. **5**). The autoencoders are trained in a self-supervised fashion by minimizing a loss based on the spectral angle divergence (SAD) (82) that measures the cosine similarity between input and reconstructed spectra.

#### Baseline methods for comparison.

We compare AE performance to conventional unmixing approaches: N-FINDR and VCA as endmember extraction algorithms followed by NNLS or FCLS to derive fractional abundances. This is performed using the RamanSPy package (83) in Python. We also compared to principal component analysis (PCA), which, despite not being designed for unmixing, is commonly used in applications. We omit the PCA results in the main text as they exhibit substantially lower performance (*SI Appendix*, Tables S2–S4 and Figs. S1 and S2).

### Benchmarking Unmixing Autoencoders on Synthetic Raman Mixtures.

We first benchmark the performance of our AE architectures on synthetic datasets created in-house.

#### Synthetic data generation.

**Fig. 2.**
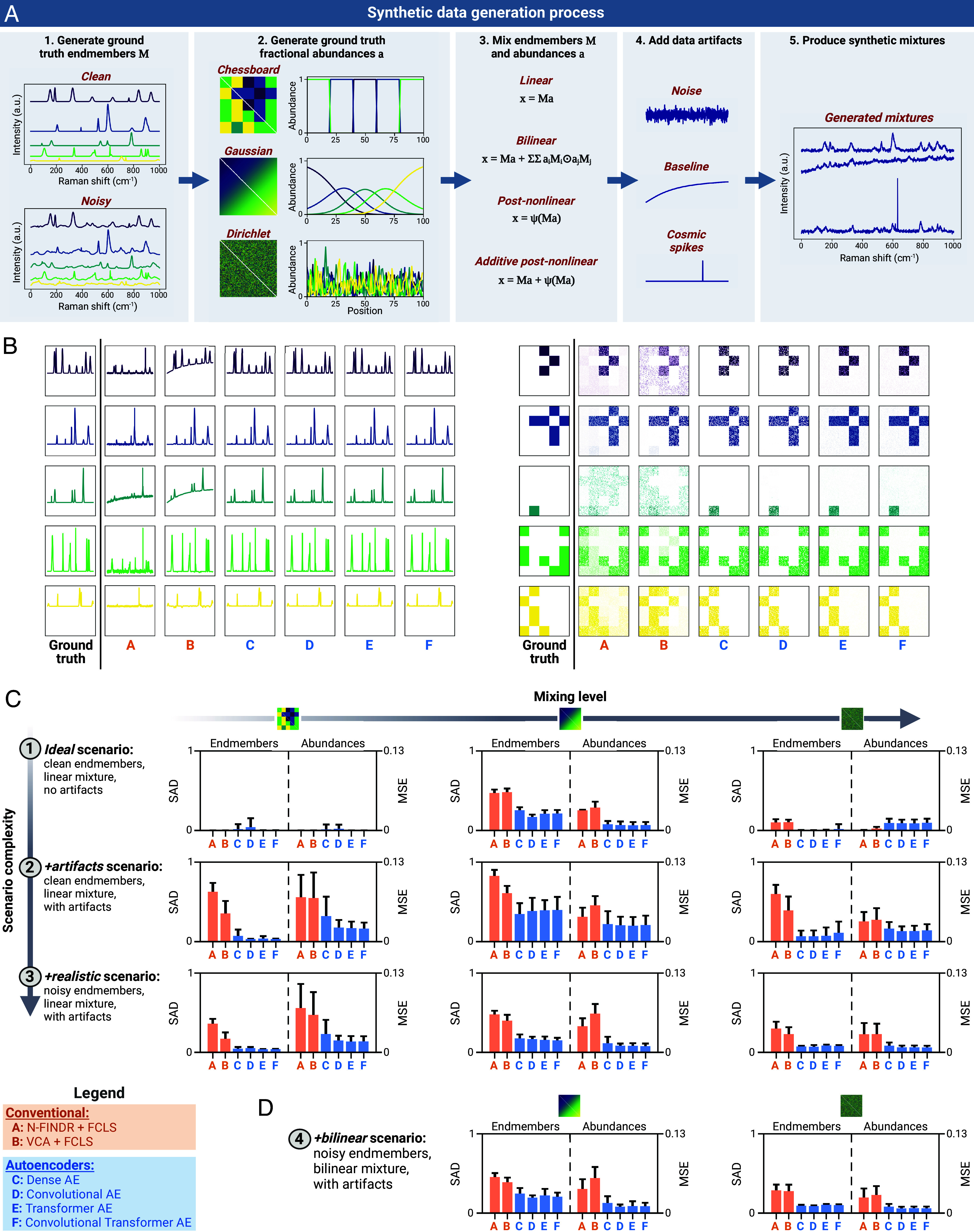
Benchmarking autoencoders on synthetic Raman mixtures. (*A*) Schematic of our synthetic data generation workflow, which allows us to create synthetic mixtures with known ground truth. (*B*) Representative unmixing results (endmembers (*Left*), fractional abundances (*Right*)) obtained by each of the six algorithms considered here when applied to one of our synthetic datasets (*Chessboard* scene, *+artifacts* scenario). (*C* and *D*) Summary of the unmixing performance of the six algorithms on 11 types of synthetic datasets, which span variable mixing levels and complexity. Linear mixtures are shown in *C*, and bilinear mixtures in *D*. CI are given by one SD around the sample mean (each bar represents n=25 evaluations: 5 datasets with 5 model repetitions each).

We developed a custom data generator that produces synthetic Raman mixtures with different characteristics (e.g., number and type of endmembers, abundance profiles, mixture model, data artifacts) with full knowledge of the ground truth endmembers and fractional abundances (Fig. 2*A*). This allows us to quantitatively compare the performance of unmixing approaches (see Fig. 2*B* for unmixing of an example synthetic dataset).

Using our data generator, we produce 11 types of synthetic datasets of variable complexity, based on four mixture scenarios over three fractional abundance scenes. In order of complexity, the four mixture scenarios are 1) a linear mixture with clean endmembers and no data artifacts (ideal); 2) a linear mixture with clean endmembers, but contaminated with artifacts representing dark noise, baseline variations, and cosmic spikes (*+artifacts*); 3) a linear mixture with noisy endmembers (i.e., containing additional smaller noise peaks) and artifacts (*+realistic*); and 4) a bilinear mixture based on the Fan model (84) with *noisy* endmembers and artifacts (*+bilinear*). For each of the four mixture scenarios, we generate three dataset variants (two for the *+bilinear* scenario since no bilinear interactions are present in our *Chessboard* scene) based on custom 100×100 fractional abundance scenes. This produces 10,000 spectra per dataset, organized into two-dimensional scenes for visualization purposes. In increasing level of mixing, we have 1) a scene comprising well-separated patches, each containing a single species (*Chessboard* scene); 2) a semimixed scene given by a Gaussian mixture of species (*Gaussian* scene); and 3) a highly mixed scene where each pixel represents a random sample of species drawn from a Dirichlet distribution (*Dirichlet* scene). Therefore, our synthetic datasets cover increasingly complex scenarios, from the *idealChessboard* dataset, which is trivial for conventional methods, to noisier, more complex mixtures containing different types of artifacts.

#### Benchmark results on linear mixtures.

We first discuss our results on the nine dataset variants created through the linear mixture scenarios (1 to 3). Such data comply with the linear mixing assumption of conventional methods, and for consistency, we equip the AE models with a decoder for linear unmixing. Fig. 2*C* summarizes the performance of the six models (two conventional and four AEs) across the nine dataset variants, with experiments performed over 5 distinct datasets and 5 model initializations for each variant (refer to *SI Appendix*, Table S2, for calculated performance metrics). We measure the discrepancy between ground truth and derived endmembers (using SAD), and the discrepancy between ground truth and derived fractional abundances (using MSE). We find that the AE models outperform the two conventional methods, providing more accurate endmembers and fractional abundances across virtually all scenarios and abundance scenes. The AEs recover the performance of the conventional methods on the simple *idealChessboard* datasets, and the improvement in AE performance becomes increasingly prominent for mixture scenarios with higher levels of noise and data artifacts.

#### Nonlinear unmixing with autoencoders.

We then proceed to our benchmark analysis on synthetic data generated using a nonlinear mixture model (i.e., *+bilinear* scenario). The results are displayed in Fig. 2*D*, where we equip our AEs with a decoder specific to the bilinear mixture model by merely adapting the decoder architecture. Again, we observe that all four AE models provide a substantial improvement in unmixing accuracy compared to methods like N-FINDR+FCLS and VCA+FCLS for both endmember and abundance estimation.

#### Computational efficiency.

**Fig. 3.**
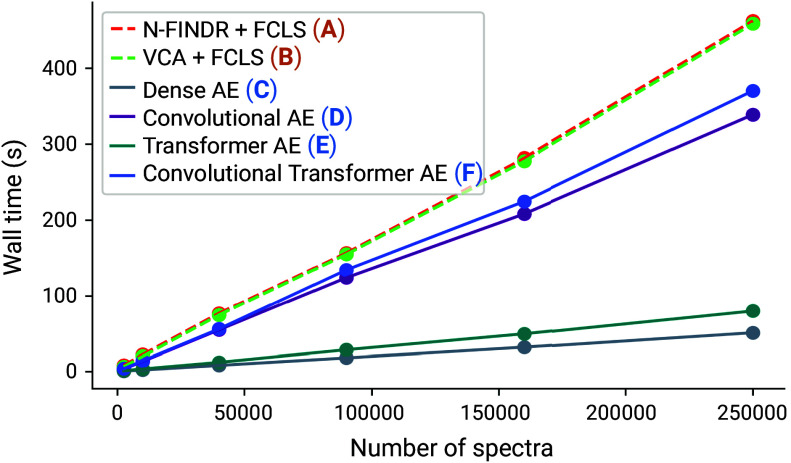
Computational efficiency analysis. The computational cost (measured as wall time) of autoencoders and conventional methods on synthetic datasets (*Chessboard* scene, *+artifacts* scenario) as the number of spectra is increased. Each dot represents the average across three evaluations (CI based on one SD are small and not visible to the eye). AE models are equipped with decoders for linear unmixing.

The computational complexity and scalability of unmixing methods can become a significant bottleneck in real-world applications, particularly for imaging and volumetric Raman scans, which can contain hundreds of thousands of spectra. To examine this issue, we profile the computational cost of our four AE methods (linear decoders) and the two conventional methods on synthetic datasets of increasing size up to 250,000 spectra. To be fair to conventional algorithms, we include the full training time for autoencoders and we use standard CPU computation to avoid any advantage from GPU acceleration. Fig. 3 shows that all AE models are faster than N-FINDR+FCLS and VCA+FCLS, which are already among the most computationally lightweight conventional unmixing techniques (85). Hence, AEs provide efficient unmixing, even without utilizing GPU acceleration and parallel processing, which can further enhance their performance.

### Validation of Unmixing Autoencoders on Experimental Raman Data from Sugar Mixtures.

**Fig. 4.**
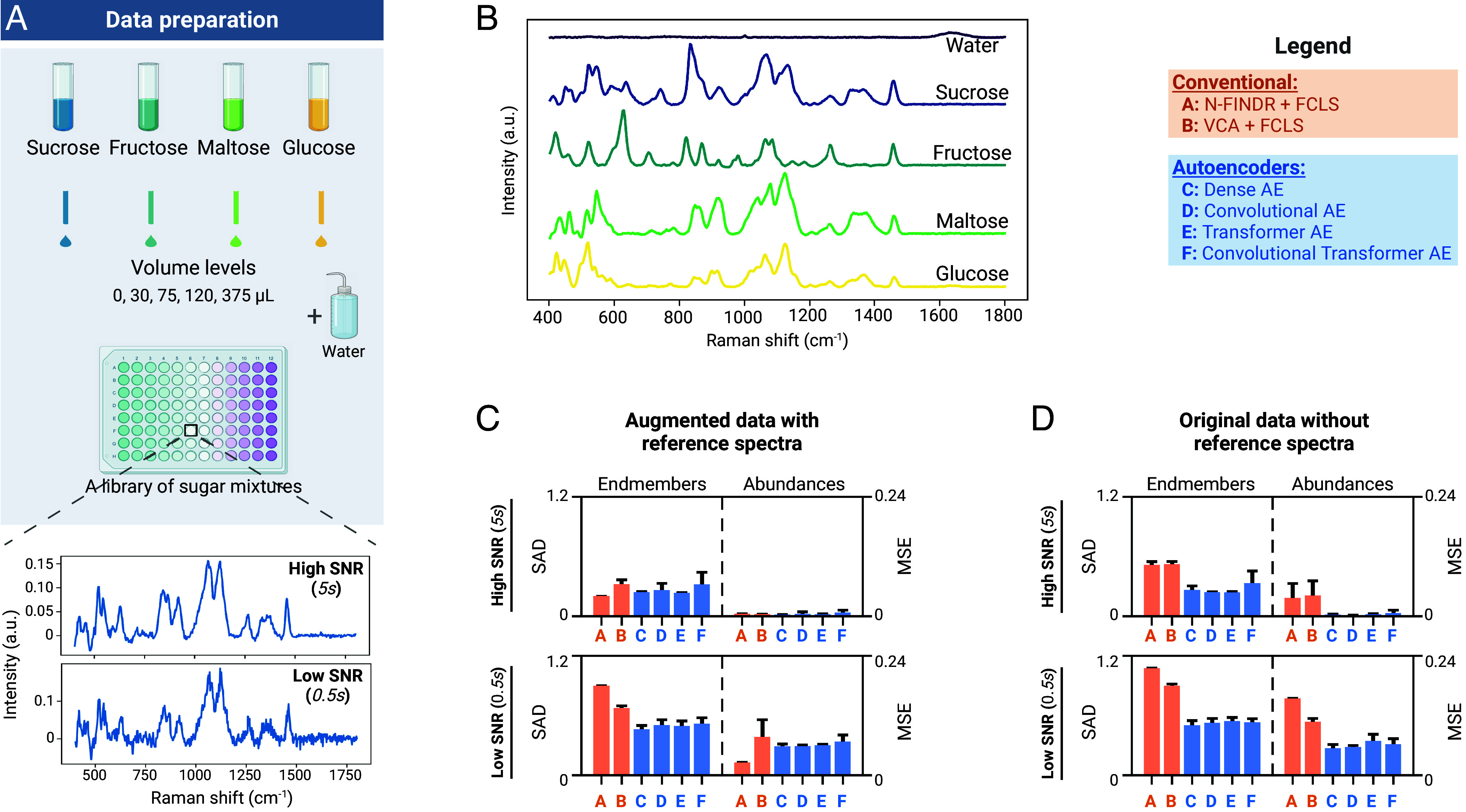
Experimental validation on Raman spectroscopy data from sugar mixtures. (*A*) Schematic diagram of sugar mixture preparation. Two sets of Raman measurements are acquired from a library of 240 sugar mixtures—high and low signal-to-noise ratio (SNR) data collected using integration times of 5s and 0.5s, respectively. (*B*) Ground-truth endmember signatures were estimated by taking the median of high SNR reference spectra measured from pure solutions containing a single sugar species. (*C* and *D*) Summary of unmixing performance for: (*C*) idealized scenario where spectra collected from pure solutions are included; and (*D*) original data without the spectra from pure solutions (i.e., no pure pixels). CI are given as one SD around the sample mean (n=5).

To validate the unmixing performance of AEs on real experimental data, we next performed benchmark analyses on data from a library of 240 sugar mixtures prepared in-house with four types of sugar (glucose, sucrose, fructose, maltose) at different concentrations (Fig. 4*A*). To evaluate different signal-to-noise (SNR) conditions, we acquired high SNR (1,920 spectra) and low SNR (7,680 spectra) measurements using a custom Raman microspectroscopy platform at integration times of 5s and 0.5s, respectively. We used these experimental datasets with ground truth to systematically evaluate unmixing algorithms under typical experimental artifacts, such as baseline shifts, environmental noise, and cosmic spikes.

We preprocess the data using RamanSPy (83) and then perform unmixing on these data to identify the content of each mixture, i.e., types of sugar and their concentrations. The ground truth is defined by the experimental concentrations and endmember signatures we obtain from reference spectra measured from five additional pure solutions (Fig. 4*B*). As with the synthetic data above, we benchmark the performance of our four AE models (linear decoders) against N-FINDR+FCLS and VCA+FCLS.

First, we consider an idealized scenario, purposefully devised to favor conventional methods, whereby endmembers are present in the data. To do this, we include in our analysis the additional reference spectra measured from pure solutions, which serve as pure pixels. When such pure pixels are available, we observe (as expected) that conventional methods (NFINDR+FCLS, VCA+FCLS) perform comparably to AEs on clean, high SNR data (Fig. 4*C*). Yet, AEs already provide improved performance in low SNR regimes.

In many experimental applications, however, the underlying endmembers are not present in the data and cannot be separately obtained (e.g., target-agnostic applications, or unknown species). To consider such cases, we analyzed our original datasets without including the reference spectra from pure solutions. Our results in Fig. 4*D* demonstrate that, in such situations, AEs substantially outperform conventional methods in both low and high SNR settings (see *SI Appendix*, Figs. S1 and S2, for additional data).

### Application of Unmixing Autoencoders to Biological Data: Volumetric Raman Imaging of a THP-1 Cell.

**Fig. 5.**
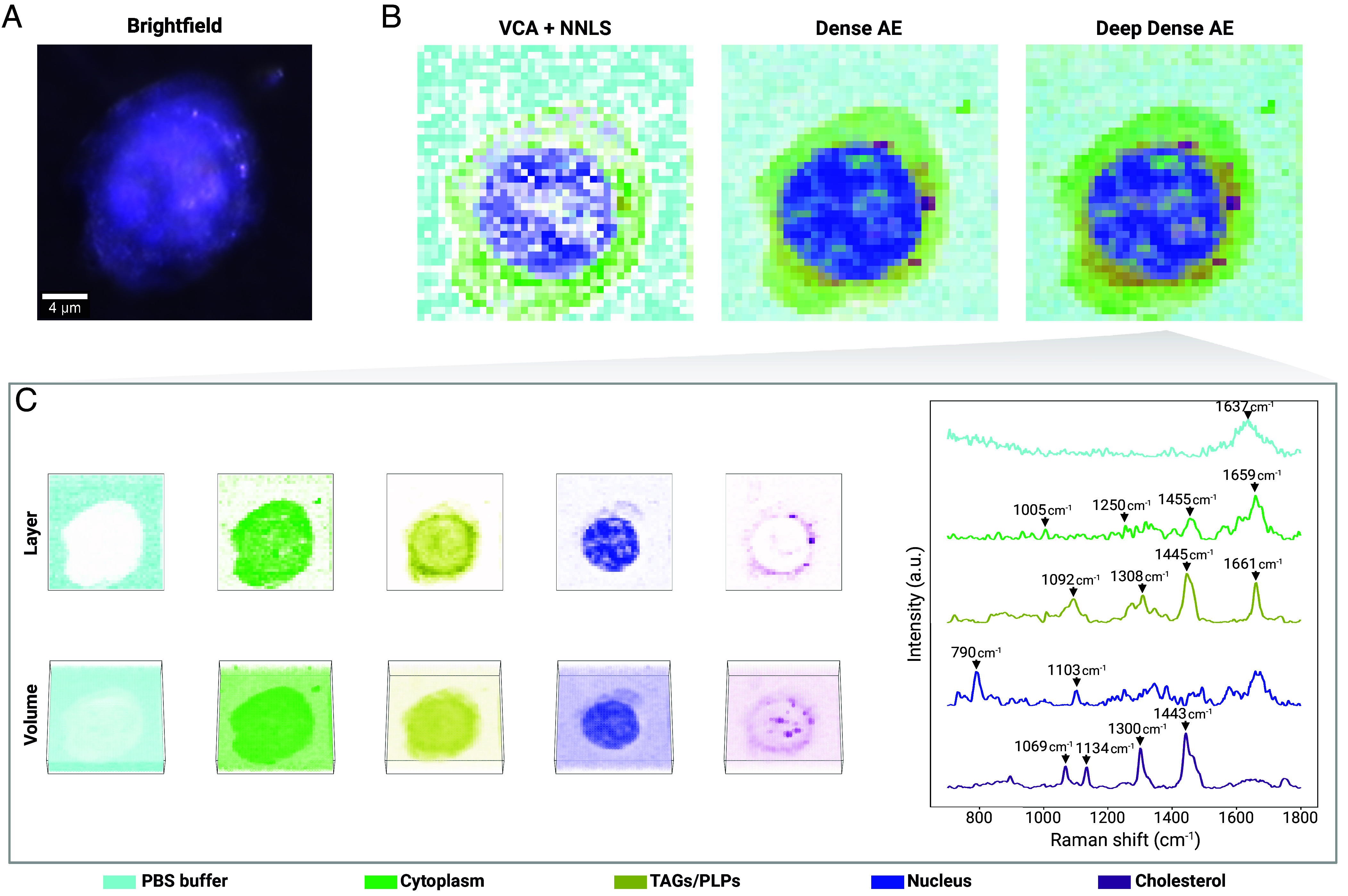
Volumetric Raman imaging of a THP-1 cell. (*A*) Brightfield image of the studied THP-1 cell. (*B*) Reconstruction of a cross-section of the cell obtained by overlaying fractional abundances derived by: VCA+NNLS, our *Dense AE* model, and our *Deep Dense AE* model. (*C*) Detailed overview of the unmixing results obtained with our *Deep Dense AE* model, displaying the spatial distribution (layer and volume) of the individual fractional abundances (*Left*) and the associated endmember signatures (*Right*). Fractional abundance maps are normalized for consistent visualization. Brightfield image and raw Raman spectroscopy data are from Kallepitis et al. (86).

As an application to biological research, we use unmixing autoencoders to analyze a low-SNR volumetric RS raster scan of a human leukemia monocytic (THP-1) cell (Fig. 5*A*) (86). Using Raman spectroscopy and chemometric techniques, the composition of the cell can be probed to study its morphology in a nondestructive, label-free manner.

After loading and preprocessing the data using RamanSPy (83), we conduct hyperspectral unmixing with: 1) VCA+NNLS—as in the original paper; 2) *Dense AE*—our simplest and most computationally efficient AE model; and 3) *Deep Dense AE*—an extension of *Dense AE* with a deeper encoder with five layers. We derive 20 endmembers, which we characterize via peak assignment to identify biochemical species present in the scanned cell, such as DNA, proteins, triglycerides (TAGs), phospholipids (PLPs) and cholesterol esters (see *Materials and Methods*).

Fig. 5*B* shows the reconstructions of the cell created by overlaying selected fractional abundances derived by each method, revealing the spatial organization of key cellular organelles, including the nucleus, cytoplasm, lipid bodies, and membranes. Although direct comparisons are challenging due to the lack of ground truth, the unmixing results of our AE models are aligned with the original findings (86), albeit with more distinct endmember signatures and well-defined abundance features (see *SI Appendix*, Figs. S3–S5, for full results). The *Deep Dense AE* model provides cleaner endmember signatures that enable more precise spectral and compositional information (Fig. 5*C*). Notably, unlike the original VCA+NNLS approach, our AEs detect cholesterol, an important functional and structural component in cells, where it plays a key role in membrane fluidity and stability, signaling pathways, and immune response (87–89).

Finally, we remark that our unsupervised autoencoder models do not utilize any ground truth information during training, in contrast with previous supervised machine learning approaches for Raman cell imaging and characterization where specific target concentrations are used as labels to train the model (e.g., using separately acquired fluorescence images) (90–93).

## Conclusion

In this work, we have presented an autoencoder-based methodology for hyperspectral unmixing in Raman spectroscopy, which we validated on a wide array of synthetic and real experimental datasets. Our results demonstrate that autoencoders are adept at handling diverse mixture scenarios and exhibit robustness against data artifacts, offering an effective, versatile, and efficient framework for RS unmixing.

The potential of autoencoders for RS unmixing opens several avenues for future research. One direction is the investigation of AE architectures with more complex decoders (66) and/or encoders [e.g., stacked and denoising AE architectures (76, 78)], as well as the use of training objectives that better capture spectral reconstruction. In particular, AE architectures that leverage spatial correlation (e.g., via spatial convolutional layers) merit further attention in imaging tasks where chemical species exhibit spatial organization. Another promising area is the use of AEs as a pretraining procedure in downstream tasks, potentially combined with other AI-based approaches [e.g., deep learning models for denoising (94)]. Building on this, the development of foundation models for Raman spectroscopy based on unmixing AEs trained on a multitude of experimental datasets would also warrant further investigation (95).

While our results demonstrate the potential of autoencoders for Raman unmixing, it is important to highlight some of the limitations. Among others, these include potentially higher data requirements for stable performance; the challenging interpretability of learned internal representations; and the sensitivity to hyperparameters requiring careful tuning. Future work should address these issues to enhance the robustness and applicability of autoencoder-based unmixing methods.

Finally, while our focus here is on RS, we wish to underscore the applicability of our work to other spectroscopic modalities, including infrared spectroscopy.

## Materials and Methods

### Hyperspectral Unmixing.

Raman spectra can be represented as vectors $x\in R_{+}^{b}$, whose components correspond to the intensity of inelastically scattered light binned over b wavelength/wavenumber bands. Raman measurements can be treated as the result of an underlying mixing of n “pure” components, defined by their Raman signatures (endmembers) $m_{i}\in R_{+}^{b},\;i=1,\ldots ,n$, and their respective proportions (fractional abundances) ${\left\{\alpha_{i}\right\}}_{i=1}^{n},\;\alpha_{i}\in R_{+}$. Hyperspectral unmixing is the inverse problem of recovering the endmembers and fractional abundances from a measurement x. The unmixing can be performed with respect to a set of known endmembers (nonblind unmixing) or without knowing the endmembers (blind unmixing). Here, we focus on blind unmixing but we also discuss how to adapt the framework to the simpler problem of nonblind unmixing.

A major hurdle for unmixing is the lack of information about the underlying mixing function. The simplest and most common model is the linear mixing model (LMM), where measurements are assumed to be a linear combination of the endmembers:

[1] $$\begin{array}{c} x=M\alpha =\sum_{i=1}^{n}\alpha_{i}m_{i}, \end{array}$$

where $M=\left[\begin{array}{cccc} m_{1} & m_{2} & \cdots & m_{n} \end{array}\right]$ is an b×n nonnegative matrix containing the n endmember signatures, and $\alpha ={\left(\alpha_{1},\alpha_{2},\cdots ,\alpha_{n}\right)}^{T}$ is an n×1 vector storing the corresponding abundances. A random noise term $\epsilon \in R^{b}$ is also often added to Eq. **1** to model stochastic variations. The abundances $\alpha_{i}$ are constrained to be nonnegative [i.e., the abundance nonnegativity constraint (ANC), $\alpha_{i}\geq 0,\forall i$], and are forced to sum to 1 when corresponding to proportions [i.e., the abundance sum-to-one constraint (ASC), ${\left||\alpha |\right|}_{1}=1$].

The linear mixing simplification allows the development of tractable approaches for unmixing, such as N-FINDR and VCA for endmember identification, and NNLS and FCLS for abundance estimation. N-FINDR and VCA are geometric methods based on the concept of a simplex in Euclidean space. N-FINDR exploits the fact that, under (Eq. **1**), endmembers represent vertices of a simplex spanning the data and operates by iteratively finding a set of points (endmembers) that maximizes the volume of the simplex they form. In contrast, VCA finds endmembers by projecting the data onto directions orthogonal to the subspace spanned by previously found endmembers and identifying new endmembers as the farthest points in these directions, effectively constructing a simplex that encompasses all data points. In both methods, the number of endmembers to extract is specified a priori by the user. Once endmember signatures M are derived, optimization-based algorithms such as NNLS and FCLS are employed to estimate the fractional abundances α for a given spectrum x by minimizing the reconstruction error between the observed data and the model $\min_{\alpha }{\left\|M\alpha -x\right\|}^{2}$. NNLS imposes the ANC, whereas FCLS imposes both the ANC and ASC.

The LMM is a good approximation when endmember species are spatially well-separated with respect to the focal volume, and complex light interactions that cause nonlinear signal contributions can be neglected (48). However, when nonlinear interactions become significant, more intricate models are required (96). To represent phenomena such as multiple scattering events, topographic variances, and shadowing effects, the LMM has been extended in remote sensing to more complex variants (96, 97), including intimate mixture models (98), bilinear models (84, 99, 100), multilinear models (101), or post-nonlinear models (102), among others. A popular bilinear mixing model is the Fan model (84):

[2] $$\begin{array}{c} x=\sum_{i=1}^{n}\alpha_{i}m_{i}+\sum_{k=1}^{n}\sum_{\begin{array}{c} l=1,\\ l\neq k \end{array}}^{n}\alpha_{k}m_{k}⊙\alpha_{l}m_{l}, \end{array}$$

where ⊙ is the Hadamard product. However, accounting for nonlinear mixing interactions increases the complexity and computational cost of unmixing (96, 97), an issue of especial relevance in Raman spectroscopy where datasets (e.g., imaging/volumetric scans) are typically larger than in remote sensing. Hence, despite its limitations, the LMM remains a cornerstone of hyperspectral unmixing in most practical settings (41, 85).

### Unmixing Autoencoders.

Consider an AE model with an encoder $E:R^{b}\to R^{m}$ that transforms spectra x into a latent space representation z=E(x) of some predefined dimension m≪b, which is subsequently passed through a decoder $D:R^{m}\to R^{b}$ to produce the output reconstruction

[3] $$\begin{array}{c} \hat{x}=D\left(z\right)=D\left(E\left(x\right)\right). \end{array}$$

Notice that the decoder D can be understood as playing the role of a mixing function on the representation z. For instance, consider a linear decoder $D_{\;\text{Lin}}$ consisting of a single linear layer defined by a b×m weight matrix W then we have

[4] $$\begin{array}{cc} \hat{x} & =D_{\;\text{Lin}}\left(z\right)=Wz\;. \end{array}$$

It follows from the formulation of the LMM (Eq. **1**) that the latent representations z resemble the abundances α, the weight matrix W resembles the matrix of endmembers M, and the dimensionality m of the latent space defines the number n of endmembers to learn.

To reinforce the physical interpretation of unmixing into the AE learning, we enforce relevant constraints, such as the nonnegativity of W and the nonnegativity (ANC) and sum-to-one constraint (ANC) of z, by applying appropriate choices of activation functions and penalties during training.

This framework can accommodate nonlinear mixture models through the design of the decoder. For instance, the bilinear Fan model (Eq. **2**) can be implemented by extending $D_{\;\text{Lin}}$ to account for the additional bilinear terms:

[5] $$\begin{array}{c} \hat{x}=D_{\;\text{Bilin}}\left(z\right)=Wz+\sum_{k=1}^{m}\sum_{\begin{array}{c} l=1,\\ l\neq k \end{array}}^{m}z_{k}w_{k}⊙z_{l}w_{l}, \end{array}$$

where $z_{k},z_{l}$ are components of z, and $w_{k},w_{l}$ are column vectors of W. Similarly, one can devise decoders suited for other mixture models (66, 79, 80), or adopt a general decoder that learns the underlying mixing model in a more data-driven manner, at the cost of interpretability of the extracted endmembers and fractional abundances. Finally, note that the AE unmixing framework can be directly adapted for nonblind unmixing by fixing the weight matrix W in the decoder to a given set of predefined endmembers.

### Autoencoder Architectures.

#### Dense AE.

This autoencoder employs an encoder comprising two fully connected (or dense) layers. The first layer projects spectra of dimension b to hidden features of dimension 128 (Leaky ReLU activation with a slope of 0.02), which the second layer further projects to latent representations of dimension n (n is the number of endmembers to extract). In the *Deep Dense AE* model used in the analysis of the THP-1 cell, we increase the number of hidden layers to five, comprising 512, 256, 128, 64, and 32 neurons, respectively, before the final layer of size n.

#### Convolutional AE.

This model extends the *Dense AE* by adding a convolutional block before the dense layers. The convolutional block consists of two layers of 1D convolutions connected in parallel, each comprising 16 filters of size 3 and 16 filters of size 5 (ReLU activation; input padded with zeroes). The outputs from these two layers are concatenated and merged (channel-wise) via a two-dimensional dense layer to produce representations of dimension b, which are then fed to the *Dense* encoder described above.

#### Transformer AE.

In this transformer-based encoder, input spectra are first projected to features of size 32 through a fully connected layer, and then fed to a transformer encoder layer comprising a multihead attention block with two attention heads of size 32 (81), followed by two fully connected layers expanding the features to size 64 (ReLU activation) and condensing back to 32 (no activation). We apply layer normalization (103) and dropout (10%) (104) after the multihead attention block and the fully connected layers. The output of the transformer block is then channeled into the last fully connected layer of size n.

#### Convolutional transformer AE.

In this model, the *Transformer AE* architecture is extended with the same convolutional block used in the *Convolutional AE*, here added before the transformer-based encoder block.

#### Decoder choice.

Our linear unmixing decoder architecture consists of a single fully connected layer using the identity activation function without bias (Eq. **4**). Our bilinear Fan decoder has the same architecture as the linear decoder but also calculates the additional bilinear interaction terms in line with Eq. **5**.

#### Physics-inspired constraints.

Fractional abundance constraints are applied through the choice of latent space activation functions. To enforce both ANC and ASC, we apply a softmax activation function in the final layer in each encoder. When only ANC is used, the activation function is changed to a “softly rectified” hyperbolic tangent function given by $\frac{1}{\gamma }log\left(1+e^{\gamma *tanh(x)}\right)$, with γ=10, which we design to ensure abundances are between 0 and 1 but do not necessarily add up to one. To ensure the nonnegativity of endmembers, we constrain the weight matrix of the linear layer in the decoder by clipping negative values to zero during training.

### Generating Synthetic Raman Mixtures.

#### Generating endmembers.

For each synthetic dataset, we first generate n endmembers spanning b spectral bands. For the scope of this work, n=5 and b=1,000. Each endmember $m_{i}\in R_{+}^{b}$ is created by a superposition of a set of $n_{\text{peaks},i}$ Gaussian peaks of different amplitudes, widths, and locations, randomly sampled as follows. The number of peaks is sampled from a discrete uniform distribution $n_{\text{peaks},i}∼U\left(5,9\right)$. Each peak p is described by $p=h_{p}\sigma_{p}\sqrt{2\pi }\;N\left(b_{p},\sigma_{p}\right)$, where N(·) represents a Gaussian distribution. The height of the peak is defined as $h_{p}=h_{1}\cdot h_{2}$, where $h_{1}=1+5\;h_{\beta }$ with $h_{\beta }∼\text{Beta}\left(1,3\right)$ and $h_{2}∼U\left(0.1,1\right)$. The center of the peak is sampled from $b_{p}∼U\left(10,b-10\right)$, and the width of the peak is defined as $\sigma_{p}=w_{p}\sigma$, with σ∼U(0.1,1).

We create two types of endmembers: clean and noisy. For the former, we produce peaks with $w_{p}=1$. For the latter, we augment clean endmembers by adding $n_{\text{peaks},i}^{\;\text{small}}∼U\left(50,99\right)$ smaller peaks sampled with $h_{1}=1/3$ and $w_{p}=2$, thus making noisy endmembers that better reflect subtle peaks and variations present in experimental Raman signatures.

#### Generating fractional abundances.

For visualization purposes, we present the fractional abundance profiles in the form of two-dimensional scenes comprising H×W pixels, where each pixel represents a fractional abundance vector $\alpha \in R_{+}^{n}$. Here, we set H=W=100, resulting in 10,000 spectra per scene/dataset. In the simplest scene (*Chessboard*), we split the scene into 20×20 square patches, each containing a single randomly assigned endmember (i.e., all 400 pixels in each patch are the same one-hot vector). Our second scene (*Gaussian*) consists of n Gaussian functions equally spaced along the diagonal of the scene. After each pixel is normalized to comply with the ASC, we obtain abundance profiles representing different levels of overlap of components. Our last fractional abundance scene (*Dirichlet*) corresponds to a highly mixed scene, where each pixel is individually sampled from a n-dimensional Dirichlet distribution, producing a random mixture of all endmembers. Note that the fractional abundance profile of each pixel in all three scenes complies with both ANC and ASC.

#### Mixing model.

Having generated a set of endmembers and an underlying fractional abundance scene, mixed data measurements $x\in R^{b}$ are created based on a mixing model chosen by the user. In this study, we consider linear mixtures (Eq. **1**) and bilinear mixtures based on the Fan model (Eq. **2**).

#### Adding data artifacts.

Finally, data artifacts (noise, baseline, cosmic spikes) can be optionally added to create more realistic synthetic Raman spectra. Here, we add Gaussian noise $\epsilon \in R^{b}$ to each spectrum, with independent and identically distributed components $\epsilon_{i}∼N\left(0,\sigma_{N}\right)$. Further, we add a baseline signal $B=h_{B}\arctan\left(\pi \left[1:b\right]/b\right)\in R^{b}$ to each spectrum with probability $p_{B}$. Finally, with probability $p_{S}$, a cosmic spike of intensity $S∼h_{S}U\left(0.75,1.25\right)$ is added to each spectrum at a band $b_{S}∼U\left\{2,b-2\right\}$. In our experiments: $\sigma_{N}=0.1$, $p_{B}=0.25$, $h_{B}=2$, $p_{S}=0.1$, $h_{S}=5$.

#### Representativeness of synthetic datasets.

To ensure that the synthetic datasets obtained with our data generator are representative, we conducted analyses comparing statistical and similarity metrics between the synthetic and experimental datasets. The results are summarized in *SI Appendix*, Figs. S6 and S7. We find that our synthetic datasets align well with the real Raman measurements (sugar solutions, cell line) across all considered metrics.

Although our synthetic data generation accounts for various sources of variability (e.g., dark noise, cosmic spikes, baseline variations), synthetic data inherently provide an idealized scenario. It is thus important to exercise caution when extrapolating findings from synthetic data alone. Nonetheless, synthetic data can serve as a valuable validation, particularly when ground truth data are unavailable or nonexistent, and provide a useful initial validation step during method development. As done here with the sugar mixtures, this validation can then be further supported by subsequent experiments using real data.

### Model Training and Evaluation.

Autoencoders were trained using the Adam optimizer (learning rate 0.001), with spectral angle distance (SAD) (82), see Eq. **7**, as a loss function between input and reconstructed spectra.

In the cases where ground-truth information is available (synthetic data and sugar mixtures), the predictive accuracy is quantified according to two measures: i) the MSE between ground-truth and predicted fractional abundances α and $\hat{\alpha }$:

[6] $$\begin{array}{c} \;\text{MSE}\left(\alpha ,\hat{\alpha }\right)=\frac{1}{n}||\alpha -\hat{\alpha }{\left|\right|}_{2}, \end{array}$$

and ii) the SAD between ground-truth and predicted endmembers $m_{i}$ and ${\hat{m}}_{i}$:

[7] $$\begin{array}{c} 0\leq \;\text{SAD}\left(m_{i},{\hat{m}}_{i}\right)=\;\arccos\left(\frac{m_{i}\cdot {\hat{m}}_{i}}{\|m_{i}{\|}_{2}\;{\left\|{\hat{m}}_{i}\right\|}_{2}}\right)\leq 1. \end{array}$$

Each evaluation is performed after first matching the derived and ground-truth endmembers (and corresponding fractional abundances) via the Hungarian algorithm with SAD as the objective to minimize. When the number of extracted endmembers n is higher than the number of ground truth endmembers $n_{\text{true}}$, we only use $n_{\text{true}}$ endmember and corresponding fractional abundance estimates to compute the performance metrics.

Note that each dataset is analyzed independently by fitting separate models from scratch.

### Analysis of Synthetic Raman Mixtures.

Each experiment on the synthetic data was performed on 5 datasets and 5 model initializations using different random seeds, resulting in 5×5=25 replicates per evaluation, or 1,650 experiments in total: 6 models (2 conventional, 4 AEs) ×11 dataset variants ×25 replicates. Random seeds were kept the same across mixture scenarios to allow direct comparison. The latent dimensionality m of each AE model is set to 5 for the *ideal* mixture scenario, and 6 for the other mixture scenarios with data artifacts. Both ANC and ASC are enforced for all experiments on synthetic data. AE models were trained for 10 epochs.

### Measuring Computational Cost.

We profile the computational cost of unmixing methods on synthetic datasets (*ideal* scenario, *Chessboard* scene) of increasing sizes, from 2,500 to 250,000 spectra. The number of endmembers to extract was set to n=5 for all methods. For each experiment, we performed three separate evaluations, measuring the wall time of each method (including the training time for autoencoders). All experiments were conducted on a MacBook Air laptop with an Apple M2 chip (8-core CPU, 10-core GPU, and 16-core Neural Engine). We only employed CPU computations to ensure a fair comparison with traditional methods which, by design, do not utilize GPU acceleration.

### Analysis of Experimental RS Data from Sugar Mixtures.

#### Preparation of sugar solutions.

We prepared 1mol/L solutions of each type of sugar (sucrose, fructose, maltose, and glucose) by dissolving the appropriate weight of sugar into 40mL of ultrapure distilled water (Invitrogen^TM^ — UltraPure^TM^ DNase/RNase-Free Distilled Water). The weights of sugars dissolved were 13.83g for sucrose (Thermo Scientific Chemicals — Sucrose, 99%), 7.279g for fructose (Thermo Scientific Chemicals — D-Fructose, 99%), 15.171g for maltose (Thermo Scientific Chemicals — D-(+)-Maltose monohydrate, 95%) and 7.279g for glucose (D-(+)-Glucose, AnalaR NORMAPUR*®* analytical reagent). All solutions were mixed and vortexed in standard 50mL centrifuge tubes until no solute was visible.

Sugar mixtures were prepared in standard 96-well plates, with a volume of 375μL per well. A full factorial experiment was performed comprising 4 volume levels for each sugar (0, 30, 75, and 120μL), filled with distilled water where necessary. Discarding the mixtures exceeding the volume of the well and the one that contains no sugar, 240 distinct mixtures were prepared. In addition, 5 extra pure solutions (i.e., 375μL of water, sucrose, fructose, maltose, or glucose) were prepared, which we used to extract reference spectra for each chemical species. This resulted in a total of 245 wells distributed in three standard 96-well plates. Mixtures were stirred using standard 200μL pipettes before spectral acquisition to ensure good mixing.

#### Raman measurements from sugar solutions.

All spectra were acquired using a custom Raman microspectroscopy platform designed for high-throughput analysis known as B-Raman. This platform is based on the Thorlabs Cerna and features the BWTek BRM-785-0.55-100-0.22-SMA laser excitation source and the Ibsen EAGLE Raman-S spectrometer. The instrument was calibrated using an Argon wavelength calibration source (AR-2 — Ocean Insight) reference lamp before data collection. The excitation wavelength was 785nm and the power incident to the samples was 36.3mW. The Raman scattering was collected in reflection via a Leica N PLAN 10x/0.25 objective with 0.25 numerical aperture. The raw spectra were acquired over the spectral wavenumber range of 142 to 3684.8 cm^−1^.

Spectra were measured from the center (horizontal) of each well at a fixed depth that was established to provide the highest signal. Two sets of data were collected from each well, at 5 s and 0.5 s integration times, to compare unmixing performance on low and high signal-to-noise ratio (SNR) data. Several measurements were collected from each well, resulting in a total of 240solutions×2measurements×4repetitions=1,920 high-SNR measurements (1,960 with reference spectra); and 240solutions×8measurements×4repetitions=7,680 low-SNR measurements (7,840 with reference spectra). Ground-truth endmembers signatures were obtained by taking the median (band-wise) of the reference spectra (8 reference spectra for each of the five components in the high-SNR setup, and 32 for each of the five components in the low-SNR setup) collected from the 5 additional wells containing pure solutions. Ground truth fractional abundances were determined by calculating the ratio of the components present in each mixture.

#### Preprocessing and analysis of sugar data.

First, we preprocess each sugar dataset: 1) cropping to the region 400 to 1,800 cm^−1^; 2) baseline correction with Adaptive Smoothness Parameter Penalized Least Squares (ASPLS) (105)—smoothing parameter $\lambda =10^{5}$, differential matrix of order 2, maximum iterations set to 100, exit criteria with tolerance t=0.001; 3) global vector normalization, where each observation is divided by the highest magnitude observed in the data. Baseline removal is important to ensure models extract relevant features (i.e., characteristic peaks) as opposed to merely capturing the trend.

To perform hyperspectral unmixing, we set the number of endmembers to extract to n=5, and we follow similar training and evaluation protocols to those employed for the synthetic data, but we increase the number of epochs to 15 for low SNR data and 50 for high SNR data given the more limited number of spectra collected. We also incorporate an additional MSE term in the training loss L of autoencoders on high SNR data:

[8] $$\begin{array}{c} L\left(x,\hat{x}\right)=\;\text{SAD}\left(x,\hat{x}\right)+\lambda \;\;\text{MSE}\left(x,\hat{x}\right), \end{array}$$

with λ=1,000. This term breaks the invariance to scale and leads to better abundance estimation given the weak water endmember (*SI Appendix*, Table S3). The standard SAD loss was used for experiments on low SNR. Each experiment is repeated for 5 model initializations.

*SI Appendix*, Table S4, presents performance evaluation using an alternative endmember distance based on Pearson’s correlation coefficient (PCC), showing an even more pronounced improvement in endmember estimation accuracy.

### Analysis of Volumetric RS Data from THP-1 Cell.

The volumetric Raman scan of the THP-1 cell (86) was collected using 0.3 s integration time and comprises a z-stack of ten 40×40 raster scans, organized into a single volumetric hypercube for analysis. We preprocess the data before unmixing using the following protocol: 1) spectral cropping to the fingerprint region 700 to 1,800 cm^−1^; 2) cosmic spike removal using the algorithm in ref. 106 with kernel of size 3 and z-value threshold of 8; 3) denoising with Savitzky–Golay filter using a cubic polynomial kernel of size 7 (107); 4) baseline correction using Asymmetric Least Squares (AsLS) with smoothing parameter $\lambda =10^{6}$, penalizing weighting factor p=0.01, differential matrix of order 2, maximum iterations set to 50, exit criteria with tolerance threshold of t=0.001 (108); 5) global MinMax normalization to the interval [0,1].

Unmixing is performed following the same AE training protocol as in other analyses, with the number of training epochs set to 20, and the number of endmembers to extract to n=20. Here, we also discard the constraint that fractional abundances must sum to one. Out of the 20 endmembers we obtain, we display the 5 deemed most biologically relevant following peak assignment as per the original paper (86). For VCA+NNLS, two of those five endmembers corresponded to the same cell organelle, namely cytoplasm, and were visualized using the same color in the merged reconstruction displayed in Fig. 5*B*. Layer visualization in Fig. 5*C* was performed on the seventh *z*-layer.

Cell organelles were determined based on the following peaks: PBS buffer - 1,637cm^−1^ (water peak); cytoplasm - 1,005cm^−1^ (phenylalanine), 1,250cm^−1^ (Amide III), 1,659cm^−1^ (Amide I) and 1,445cm^−1^ (CH deformations of proteins and lipids); TAGs/PLPs - 1,092cm^−1^ (C–C stretching), 1,308cm^−1^ (CH_2_ twists), 1,445cm^−1^ (CH deformation), and 1,661cm^−1^ (C=C stretching); nucleus/DNA - 790cm^−1^ (symmetric phosphodiester stretch and ring breathing modes of pyrimidine bases) and 1,103cm^−1^ (symmetric dioxy-stretch of the phosphate backbone); cholesterol - 1,069cm^−1^ and 1,134cm^−1^ (cholesteryl stearate), 1,300cm^−1^ (CH_2_ twists), and 1,443cm^−1^ (CH deformation) (1, 86, 109).

### Implementation.

We conducted our analyses in Python, using TensorFlow (110) for autoencoder model development and training, and the RamanSPy package (83) for unmixing with conventional methods, data loading and management, preprocessing, and plotting.

## Supplementary Material

Appendix 01 (PDF)

## Data Availability

The original data sets presented in this work, both synthetic data and experimental data from sugar mixtures, are available at https://doi.org/10.5281/zenodo.10779223 (111). Previously published data from a THP-1 cell used in this work is available at https://doi.org/10.5281/zenodo.256329 (scan ‘001’) (86). The code and associated scripts developed for this work are available at https://github.com/barahona-research-group/raman-unmixing-aes (112). The synthetic data generator has also been integrated into the RamanSPy Python package (https://github.com/barahona-research-group/RamanSPy) (83).
