# Supplementary material for: Hyperspectral unmixing for Raman spectroscopy via physics-constrained autoencoders
Source: Proc Natl Acad Sci U S A. 2024 Oct 29;121(45):e2407439121. doi: 10.1073/pnas.2407439121 (PMC11551349; doi:10.1073/pnas.2407439121)
Supplement: Supplementary file 1 — Appendix 01 (PDF) [file pnas.2407439121.sapp.pdf]

# Supporting Information for

## Hyperspectral unmixing for Raman spectroscopy via physics-constrained autoencoders

**Dimitar Georgiev, Álvaro Fernández-Galiana, Simon Vilms Pedersen, Georgios Papadopoulos, Ruoxiao Xie, Molly M. Stevens, Mauricio Barahona**

**Molly M. Stevens & Mauricio Barahona.**
**E-mail: molly.stevens@dpag.ox.ac.uk & m.barahona@imperial.ac.uk**

### This PDF file includes:

Figs. S1 to S7
Tables S1 to S4

**Table S1. Comparative analysis of autoencoders and standard unmixing techniques.**

| | Standard unmixing methods (e.g., VCA+NNLS) | Autoencoders |
|---|---|---|
| Assumptions | Standard methods rely on strict assumptions - e.g. endmembers present in the data as 'pure pixels'; fixed mixture model; number of endmembers is known. | Autoencoders can facilitate data-driven unmixing without many strong assumptions about the data and task. |
| Mixture model | A specific model is assumed *a priori*. Usually, a linear mixing only, as alternatives can become too complex and computationally expensive. | Autoencoders can be designed to capture various mixture models, including non-linear mixtures. This can be enforced by appropriate architectural constraints or learned in a data-driven manner. |
| Input modality & capturing spatial correlations | Spectra are processed individually. If imaging or volumetric data are provided, these are typically unfolded and used pixel-wise, thereby discarding any spatial correlations. | Autoencoders can be extended to utilize the spatial correlations available in imaging or volumetric data by incorporating (2D, 3D, and/or 4D) convolutional layers. |
| Simultaneously deriving endmembers and fractional abundances? | Typically, two separate algorithms are applied. | Autoencoders simultaneously extract both endmembers and fractional abundances by default. They can also be adjusted for non-blind unmixing by fixing a pre-defined endmember matrix in the decoder. |
| Robustness to non-specific signals and preprocessing – e.g. noise, baseline | Performance is usually extremely dependent on the quality of the data, as well as on preprocessing, which can vary greatly from application to application. | Autoencoders can incorporate feature selection blocks and training loss to promote invariance to scaling, baselines, and noise, making them more robust to artifacts and outliers. |
| Variable spectral axis | Observations are assumed to share a common spectral axis, and the spectral axis is discarded. This impedes the integration of data across experimental setups unless they share the same spectral axis. | The axis can be integrated as an input to the model (e.g. as a positional encoding), allowing the direct integration of diverse data sources. |
| Scalability | Even the simplest models can become computationally prohibitive for larger datasets. More advanced options are intractable in real-world applications involving imaging or volumetric Raman scans. | Deep neural networks are designed to be scalable and parallelizable out-of-the-box. |
| Number of endmembers | Typically, fixed *a priori*. | Normally fixed *a priori*, too, but can potentially be learned by introducing sparsity and information-theoretic criteria in the latent space, for instance. |
| Extensibility | Most conventional techniques are based on specialized optimization-based algorithms tailored for the particular unmixing task (e.g. mixture model, scene type, number of endmembers), which makes their extension challenging. | Easy extension and adaptation. One can readily expand unmixing autoencoders into more advanced variants, as well as integrate models into established AI and ML pipelines. |
| Downstream applications | Conventional techniques are generally constrained within hyperspectral unmixing, with algorithms only outputting the derived unmixing results. | Autoencoders can be used as feature extractors in a variety of modeling and predictive downstream tasks, such as classification, clustering and anomaly detection. They can also leverage transfer learning techniques to improve performance in situations with limited labeled data by utilizing unmixing autoencoders for self-supervised pre-training. |
| Data requirements | Standard methods typically require the presence of endmember signatures as 'pure pixels' in the dataset, and their effectiveness depends on the availability of these signatures. | AEs do not necessarily require 'pure pixel' signatures but need large datasets with representative mixtures for effective learning, which might not always be available or easily obtainable. |
| Interpretability | Standard methods are based on explicit mathematical models, making the results generally interpretable and traceable back to the original input features. | The internal representations learned by AEs are typically challenging to interpret and can represent complex, abstract relationships that are challenging to reverse-engineer or directly correlate with physical properties. |
| Hyperparameters | The primary hyperparameter in standard methods is the number of endmember signatures to consider, which simplifies the tuning process. | AEs model training can involve numerous hyperparameters (e.g., number of layers, size of layers, learning rate, training epochs, batch size), requiring extensive tuning and optimization for effective performance. |

**Table S2. Benchmark results on diverse synthetic Raman mixture datasets.** Each value represents the average result of 25 replicates, including 5 dataset and 5 model initialisations. Confidence intervals are calculated as one standard deviation around the sample mean. Values rounded to 3 decimal places. Best and second best results (mean value) are given in **underlined bold** and **bold**, respectively.

| | | *Chessboard* | | *Gaussian* | | *Dirichlet* | |
|---|---|---|---|---|---|---|---|
| | Method | Endmembers (SAD) ↓ | Abundances (MSE) ↓ | Endmembers (SAD) ↓ | Abundances (MSE) ↓ | Endmembers (SAD) ↓ | Abundances (MSE) ↓ |
| *ideal* | PCA | 0.874 ± 0.139 | 22.592 ± 8.969 | 1.051 ± 0.081 | 5.387 ± 1.876 | 0.847 ± 0.092 | 3.811 ± 1.291 |
| | N-FINDR + FCLS | **0.000** ± **0.000** | **0.000** ± **0.000** | 0.473 ± 0.042 | 0.033 ± 0.001 | 0.101 ± 0.040 | **0.001** ± **0.000** |
| | VCA + FCLS | **0.000** ± **0.000** | **0.000** ± **0.000** | 0.482 ± 0.047 | 0.038 ± 0.009 | 0.105 ± 0.031 | **0.003** ± **0.003** |
| | Dense AE | 0.015 ± 0.065 | 0.002 ± 0.008 | 0.255 ± 0.037 | **0.010** ± **0.005** | **0.003** ± **0.001** | 0.012 ± 0.007 |
| | Convolutional AE | 0.042 ± 0.111 | 0.002 ± 0.007 | **0.168** ± **0.033** | **0.009** ± **0.006** | **0.003** ± **0.001** | 0.012 ± 0.006 |
| | Transformer AE | **0.002** ± **0.000** | **0.000** ± **0.000** | **0.212** ± **0.043** | **0.009** ± **0.005** | **0.007** ± **0.002** | 0.012 ± 0.006 |
| | Convolutional Transformer AE | **0.002** ± **0.000** | **0.000** ± **0.000** | 0.215 ± 0.042 | **0.009** ± **0.005** | 0.017 ± 0.062 | 0.013 ± 0.007 |
| *+ artifacts* | PCA | 0.819 ± 0.072 | 79.38 ± 49.727 | 1.009 ± 0.082 | 61.488 ± 48.586 | 0.869 ± 0.069 | 60.154 ± 48.330 |
| | N-FINDR + FCLS | 0.629 ± 0.111 | 0.073 ± 0.036 | 0.828 ± 0.075 | 0.041 ± 0.015 | 0.599 ± 0.116 | 0.033 ± 0.015 |
| | VCA + FCLS | 0.353 ± 0.159 | 0.071 ± 0.042 | 0.609 ± 0.093 | 0.059 ± 0.015 | 0.392 ± 0.175 | 0.036 ± 0.019 |
| | Dense AE | 0.072 ± 0.081 | 0.042 ± 0.032 | **0.347** ± **0.137** | 0.029 ± 0.021 | **0.068** ± **0.070** | 0.021 ± 0.011 |
| | Convolutional AE | **0.033** ± **0.003** | 0.023 ± 0.013 | **0.385** ± **0.166** | **0.027** ± **0.014** | **0.068** ± **0.072** | **0.017** ± **0.008** |
| | Transformer AE | **0.039** ± **0.031** | **0.022** ± **0.011** | 0.393 ± 0.129 | **0.026** ± **0.014** | **0.073** ± **0.105** | **0.017** ± **0.009** |
| | Convolutional Transformer AE | **0.033** ± **0.004** | **0.021** ± **0.010** | 0.399 ± 0.164 | **0.027** ± **0.015** | 0.112 ± 0.140 | **0.019** ± **0.010** |
| *+ realistic* | PCA | 0.967 ± 0.074 | 88.132 ± 50.053 | 1.079 ± 0.045 | 63.735 ± 48.513 | 0.993 ± 0.089 | 61.244 ± 48.733 |
| | N-FINDR + FCLS | 0.361 ± 0.062 | 0.072 ± 0.040 | 0.478 ± 0.047 | 0.043 ± 0.013 | 0.299 ± 0.087 | 0.030 ± 0.019 |
| | VCA + FCLS | 0.173 ± 0.079 | 0.061 ± 0.038 | 0.400 ± 0.075 | 0.063 ± 0.016 | 0.229 ± 0.088 | 0.030 ± 0.017 |
| | Dense AE | 0.045 ± 0.024 | 0.030 ± 0.023 | 0.177 ± 0.050 | 0.015 ± 0.011 | **0.073** ± **0.012** | **0.011** ± **0.006** |
| | Convolutional AE | 0.054 ± 0.013 | **0.019** ± **0.008** | 0.168 ± 0.030 | **0.011** ± **0.004** | **0.071** ± **0.022** | **0.008** ± **0.003** |
| | Transformer AE | **0.039** ± **0.005** | **0.018** ± **0.009** | **0.156** ± **0.044** | **0.011** ± **0.005** | 0.085 ± 0.014 | **0.008** ± **0.003** |
| | Convolutional Transformer AE | **0.040** ± **0.005** | **0.018** ± **0.008** | **0.151** ± **0.033** | **0.010** ± **0.004** | 0.081 ± 0.012 | **0.008** ± **0.003** |
| *+ bilinear* | PCA | —"— | —"— | 1.117 ± 0.071 | 67.189 ± 48.404 | 1.038 ± 0.112 | 63.915 ± 48.423 |
| | N-FINDR + FCLS | —"— | —"— | 0.456 ± 0.049 | 0.039 ± 0.016 | 0.287 ± 0.074 | 0.025 ± 0.015 |
| | VCA + FCLS | —"— | —"— | 0.391 ± 0.058 | 0.057 ± 0.018 | 0.277 ± 0.082 | 0.030 ± 0.015 |
| | Dense AE (bilinear) | —"— | —"— | 0.247 ± 0.070 | 0.017 ± 0.011 | **0.094** ± **0.011** | **0.010** ± **0.005** |
| | Convolutional AE (bilinear)* | —"— | —"— | ***0.194*** ± **0.030** | **0.010** ± **0.004** | **0.087** ± **0.010** | **0.008** ± **0.003** |
| | Transformer AE (bilinear) | —"— | —"— | 0.222 ± 0.084 | 0.012 ± 0.008 | 0.105 ± 0.012 | **0.008** ± **0.003** |
| | Convolutional Transformer AE (bilinear) | —"— | —"— | **0.208** ± **0.051** | **0.011** ± **0.006** | 0.100 ± 0.010 | **0.008** ± **0.003** |

**Table S3. Full unmixing results on Raman spectroscopy data from sugar solutions.** Each value represents the average result of 5 replicates. Confidence intervals are calculated as one standard deviation around the sample mean. Values rounded to 3 decimal places. Best and second best results (mean value) are given in **underlined bold** and **bold**, respectively.

| | | *High SNR* | | | | *Low SNR* | | | |
|---|---|---|---|---|---|---|---|---|---|
| | | *with endmembers* | | *without endmembers* | | *with endmembers* | | *without endmembers* | |
| | Method | Endmembers (SAD) ↓ | Abundances (MSE) ↓ | Endmembers (SAD) ↓ | Abundances (MSE) ↓ | Endmembers (SAD) ↓ | Abundances (MSE) ↓ | Endmembers (SAD) ↓ | Abundances (MSE) ↓ |
| | PCA | 1.018 ± 0.000 | 0.073 ± 0.000 | 1.026 ± 0.000 | 0.072 ± 0.000 | 1.173 ± 0.000 | 0.075 ± 0.000 | 1.210 ± 0.000 | 0.073 ± 0.000 |
| | N-FINDR+FCLS | **0.202** ± **0.000** | 0.005 ± 0.000 | 0.515 ± 0.035 | 0.037 ± 0.029 | 0.900 ± 0.000 | **0.026** ± **0.000** | 1.077 ± 0.000 | 0.154 ± 0.000 |
| | VCA+FCLS | 0.322 ± 0.044 | **0.004** ± **0.000** | 0.525 ± 0.026 | 0.042 ± 0.029 | 0.677 ± 0.022 | 0.077 ± 0.035 | 0.901 ± 0.017 | 0.108 ± 0.006 |
| *SAD* | Dense AE | 0.212 ± 0.004 | 0.057 ± 0.001 | 0.219 ± 0.001 | 0.058 ± 0.001 | **0.462** ± **0.038** | **0.058** ± **0.005** | **0.503** ± **0.046** | **0.054** ± **0.007** |
| | Convolutional AE | **0.203** ± **0.001** | 0.058 ± 0.001 | **0.215** ± **0.002** | 0.058 ± 0.001 | 0.503 ± 0.055 | **0.058** ± **0.002** | 0.529 ± 0.041 | **0.057** ± **0.003** |
| | Transformer AE | 0.206 ± 0.001 | 0.058 ± 0.001 | **0.218** ± **0.002** | 0.028 ± 0.002 | **0.496** ± **0.050** | 0.061 ± 0.002 | 0.545 ± 0.037 | 0.069 ± 0.013 |
| | Conv. Trans. AE | 0.208 ± 0.002 | 0.057 ± 0.001 | **0.218** ± **0.001** | 0.057 ± 0.001 | 0.521 ± 0.059 | 0.068 ± 0.013 | 0.533 ± 0.035 | 0.062 ± 0.011 |
| *MSE + SAD* | Dense AE | 0.242 ± 0.008 | **0.003** ± **0.001** | 0.265 ± 0.040 | **0.003** ± **0.002** | 0.498 ± 0.038 | 0.070 ± 0.006 | **0.509** ± **0.045** | 0.069 ± 0.008 |
| | Convolutional AE | 0.264 ± 0.068 | **0.004** ± **0.004** | 0.240 ± 0.007 | **0.002** ± **0.000** | 0.568 ± 0.057 | 0.083 ± 0.017 | 0.593 ± 0.052 | 0.080 ± 0.013 |
| | Transformer AE | 0.234 ± 0.006 | **0.004** ± **0.001** | 0.240 ± 0.009 | 0.004 ± 0.001 | 0.606 ± 0.034 | 0.101 ± 0.026 | 0.632 ± 0.014 | 0.097 ± 0.028 |
| | Conv. Trans. AE | 0.321 ± 0.121 | 0.008 ± 0.005 | 0.333 ± 0.123 | 0.007 ± 0.006 | 0.570 ± 0.068 | 0.113 ± 0.012 | 0.665 ± 0.179 | 0.164 ± 0.037 |

**Table S4. Full unmixing results on Raman spectroscopy data from sugar solutions with respect to an alternative endmember similarity metric PCC based on the Pearson correlation coefficient.** Each value represents the average result of 5 replicates. Confidence intervals are calculated as one standard deviation around the sample mean. Values rounded to 3 decimal places. Best and second best results (mean value) are given in **underlined bold** and **bold**, respectively. The PCC between two endmember signatures $x$ and $y$ is defined as $\mathrm{PCC}(x,y) = 1 - \frac{\sum(x_i - \bar{x})\sum(y_i - \bar{y})}{\sqrt{\sum(x_i - \bar{x})^2 \sum(y_i - \bar{y})^2}}$.

| | | *High SNR* | | | | *Low SNR* | | | |
|---|---|---|---|---|---|---|---|---|---|
| | | *with endmembers* | | *without endmembers* | | *with endmembers* | | *without endmembers* | |
| | Method | Endmembers (PCC) ↓ | Abundances (MSE) ↓ | Endmembers (PCC) ↓ | Abundances (MSE) ↓ | Endmembers (PCC) ↓ | Abundances (MSE) ↓ | Endmembers (PCC) ↓ | Abundances (MSE) ↓ |
| | PCA | 0.547 ± 0.000 | 0.073 ± 0.000 | 0.541 ± 0.000 | 0.072 ± 0.000 | 0.641 ± 0.000 | 0.075 ± 0.000 | 0.691 ± 0.000 | 0.073 ± 0.000 |
| | N-FINDR+FCLS | 0.039 ± 0.000 | 0.005 ± 0.000 | 0.203 ± 0.055 | 0.037 ± 0.029 | 0.520 ± 0.000 | <u>**0.026**</u> ± <u>**0.000**</u> | 0.641 ± 0.000 | 0.154 ± 0.000 |
| | VCA+FCLS | 0.099 ± 0.047 | **0.004** ± **0.000** | 0.221 ± 0.056 | 0.042 ± 0.029 | 0.325 ± 0.019 | 0.077 ± 0.035 | 0.482 ± 0.005 | 0.108 ± 0.006 |
| *SAD* | Dense AE | 0.029 ± 0.001 | 0.057 ± 0.001 | **0.031** ± **0.000** | 0.058 ± 0.001 | <u>**0.164**</u> ± <u>**0.042**</u> | **0.058** ± **0.005** | **0.198** ± **0.046** | <u>**0.054**</u> ± <u>**0.007**</u> |
| | Convolutional AE | <u>**0.027**</u> ± <u>**0.000**</u> | 0.058 ± 0.001 | <u>**0.030**</u> ± <u>**0.002**</u> | 0.058 ± 0.001 | 0.207 ± 0.052 | **0.058** ± **0.002** | 0.228 ± 0.030 | **0.057** ± **0.003** |
| | Transformer AE | **0.028** ± **0.000** | 0.058 ± 0.001 | **0.031** ± **0.000** | 0.028 ± 0.002 | 0.200 ± 0.048 | 0.061 ± 0.002 | 0.243 ± 0.032 | 0.069 ± 0.013 |
| | Conv. Trans. AE | **0.028** ± **0.000** | 0.057 ± 0.001 | **0.031** ± **0.000** | 0.057 ± 0.001 | 0.226 ± 0.059 | 0.068 ± 0.013 | 0.236 ± 0.034 | 0.062 ± 0.011 |
| *MSE + SAD* | Dense AE | 0.037 ± 0.002 | <u>**0.003**</u> ± <u>**0.001**</u> | 0.047 ± 0.018 | **0.003** ± **0.002** | **0.174** ± **0.032** | 0.070 ± 0.006 | <u>**0.185**</u> ± <u>**0.042**</u> | 0.069 ± 0.008 |
| | Convolutional AE | 0.056 ± 0.044 | **0.004** ± **0.004** | 0.037 ± 0.002 | <u>**0.002**</u> ± <u>**0.000**</u> | 0.243 ± 0.044 | 0.083 ± 0.017 | 0.272 ± 0.040 | 0.080 ± 0.013 |
| | Transformer AE | 0.035 ± 0.002 | **0.004** ± **0.001** | 0.037 ± 0.002 | 0.004 ± 0.001 | 0.296 ± 0.041 | 0.101 ± 0.026 | 0.311 ± 0.020 | 0.097 ± 0.028 |
| | Conv. Trans. AE | 0.104 ± 0.096 | 0.008 ± 0.005 | 0.108 ± 0.099 | 0.007 ± 0.006 | 0.250 ± 0.072 | 0.113 ± 0.012 | 0.348 ± 0.176 | 0.164 ± 0.037 |

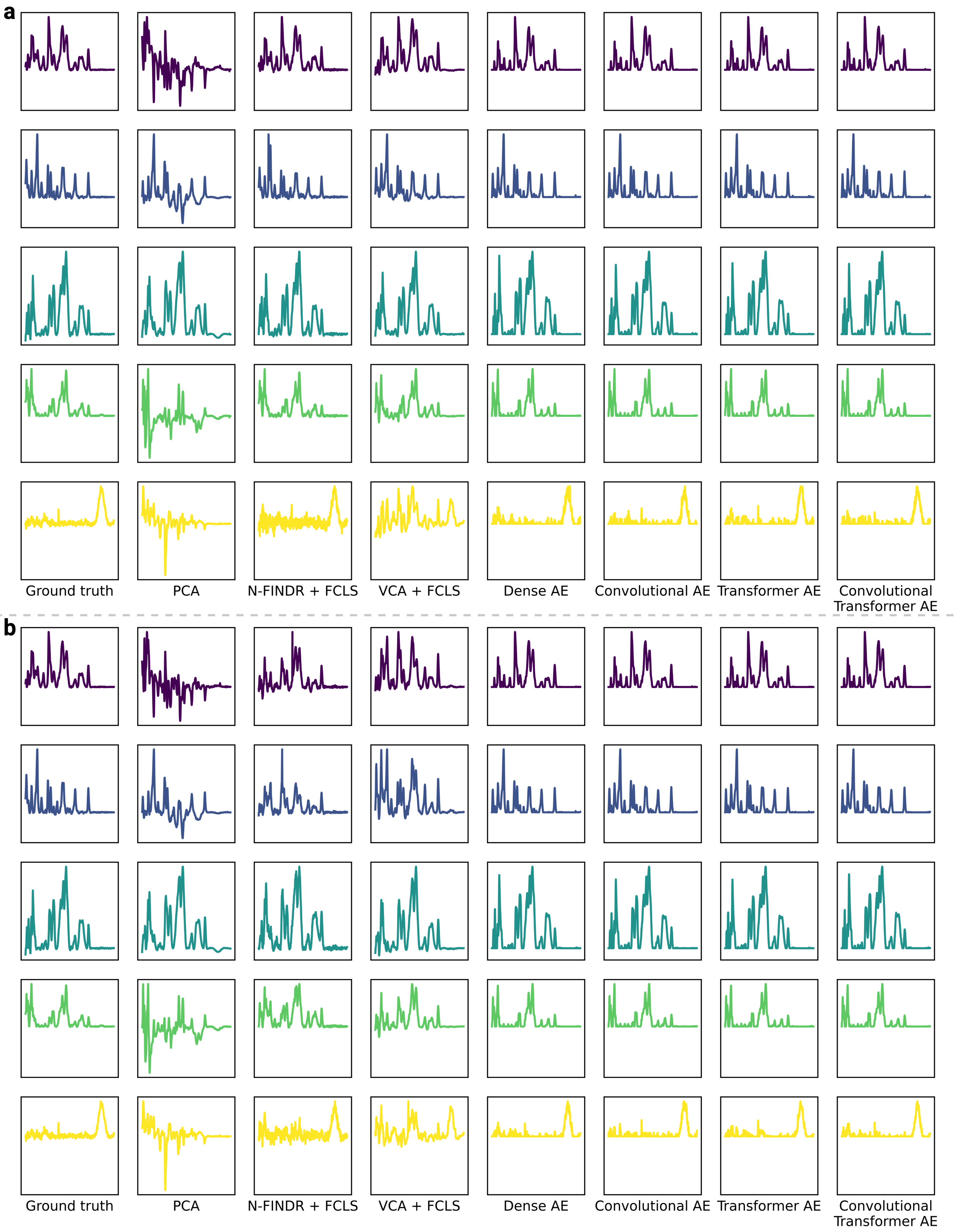


**Fig. S1. Endmember estimates on high SNR sugar data.** Qualitative comparison of derived endmembers on the high SNR sugar datasets - with (**a**), and without reference spectra (**b**). Endmembers are scaled such that their maximum intensity is equal to $1$ for visualization purposes. *x*-axes represent the Raman shift region $400\text{–}1800\,\mathrm{cm}^{-1}$, and *y*-axes, which are shared for each row, represents normalized intensity (a.u.).

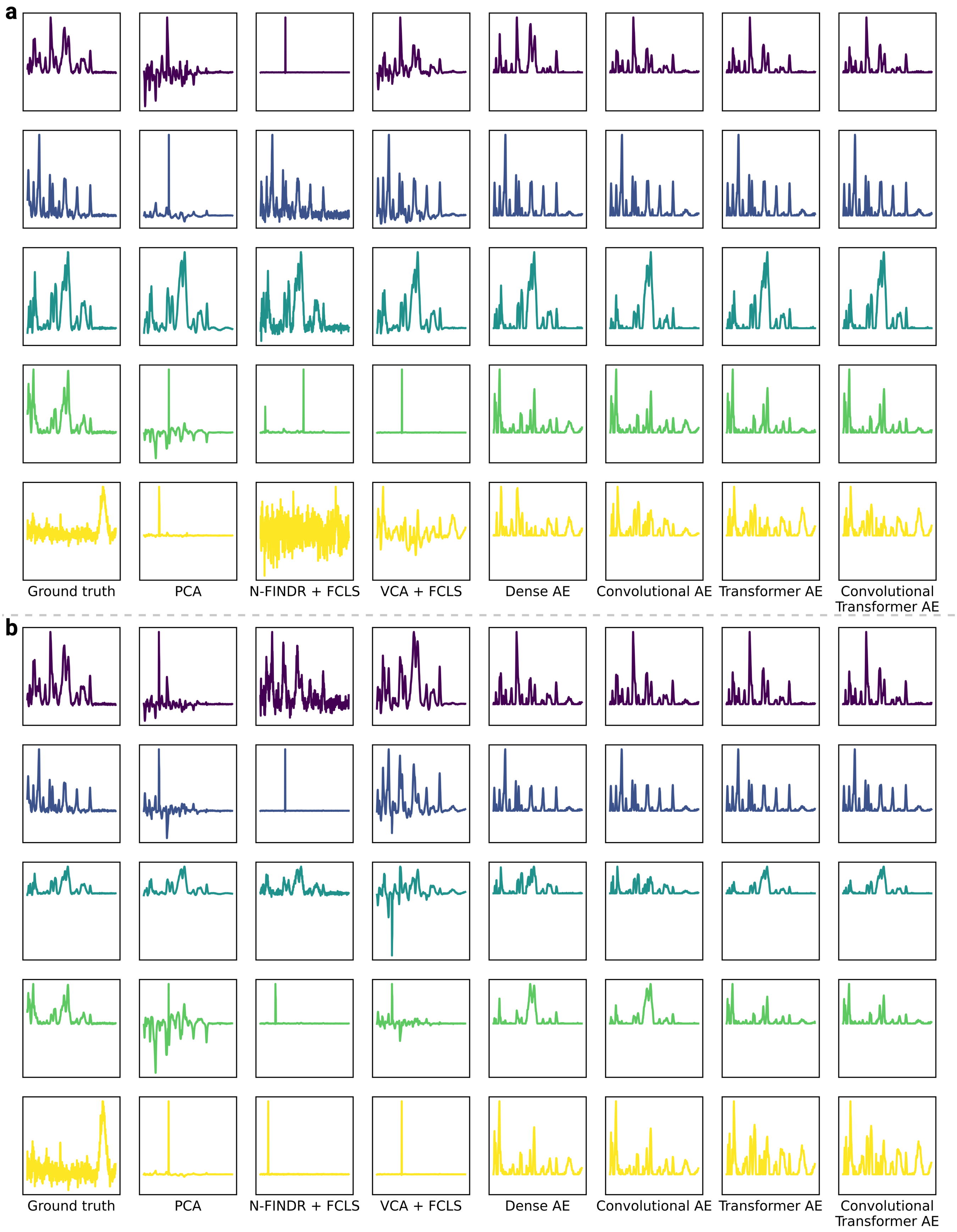


**Fig. S2. Endmember estimates on low SNR sugar data.** Qualitative comparison of derived endmembers on the low SNR sugar datasets - with (**a**), and without reference spectra (**b**). Endmembers are scaled such that their maximum intensity is equal to $1$ for visualization purposes. $x$-axes represent the Raman shift region $400$–$1800\,\mathrm{cm}^{-1}$, and $y$-axes, which are shared for each row, represents normalized intensity (a.u.).

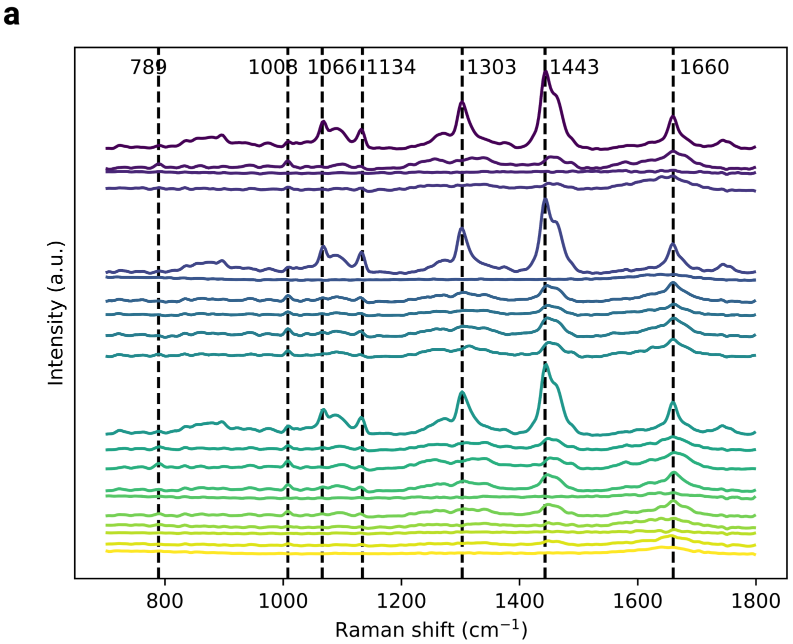


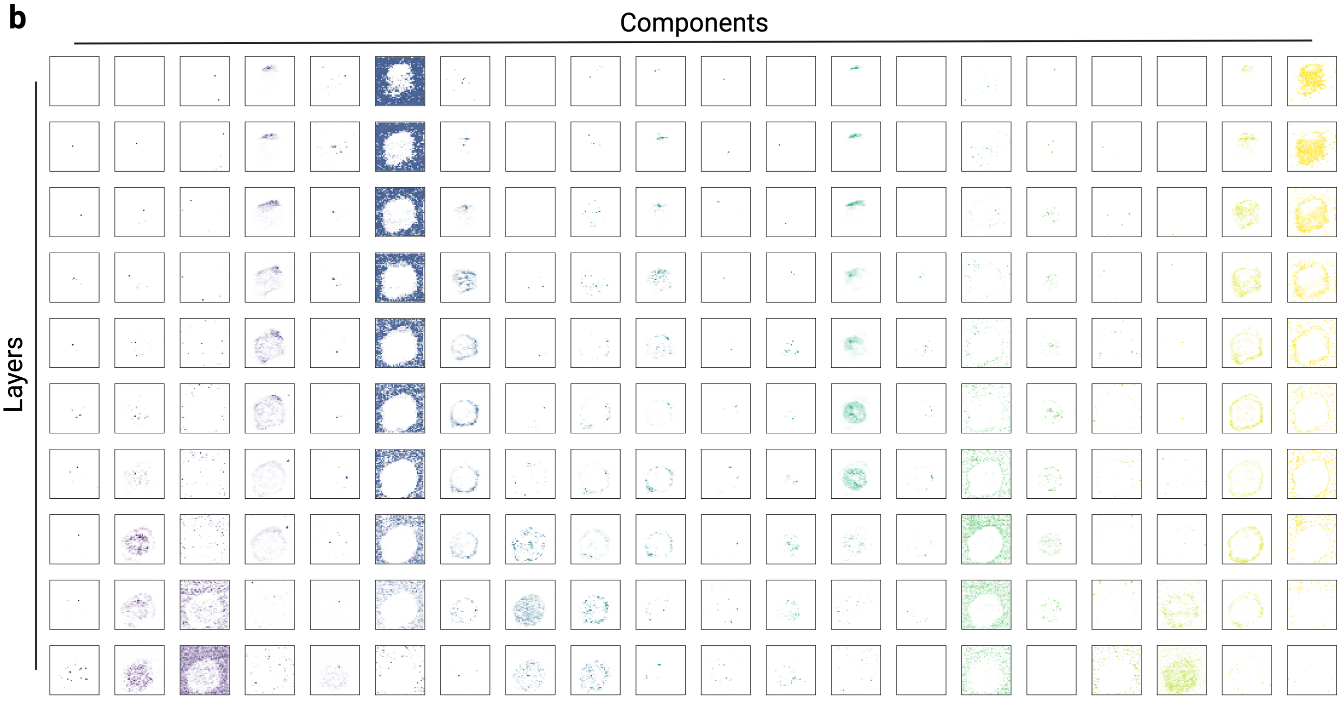


**Fig. S3. Full unmixing results obtained with VCA + NNLS on the THP-1 cell data. a,** Derived endmembers. **b,** Derived fractional abundances.

**a**

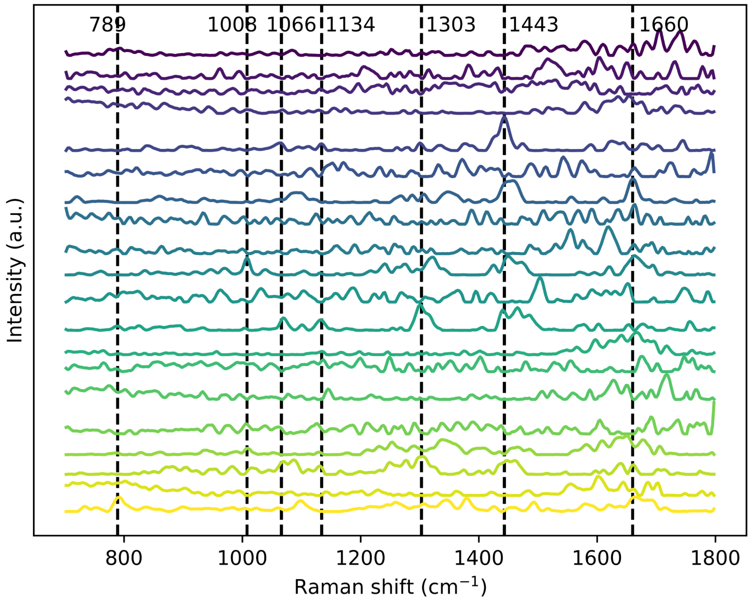


**b**

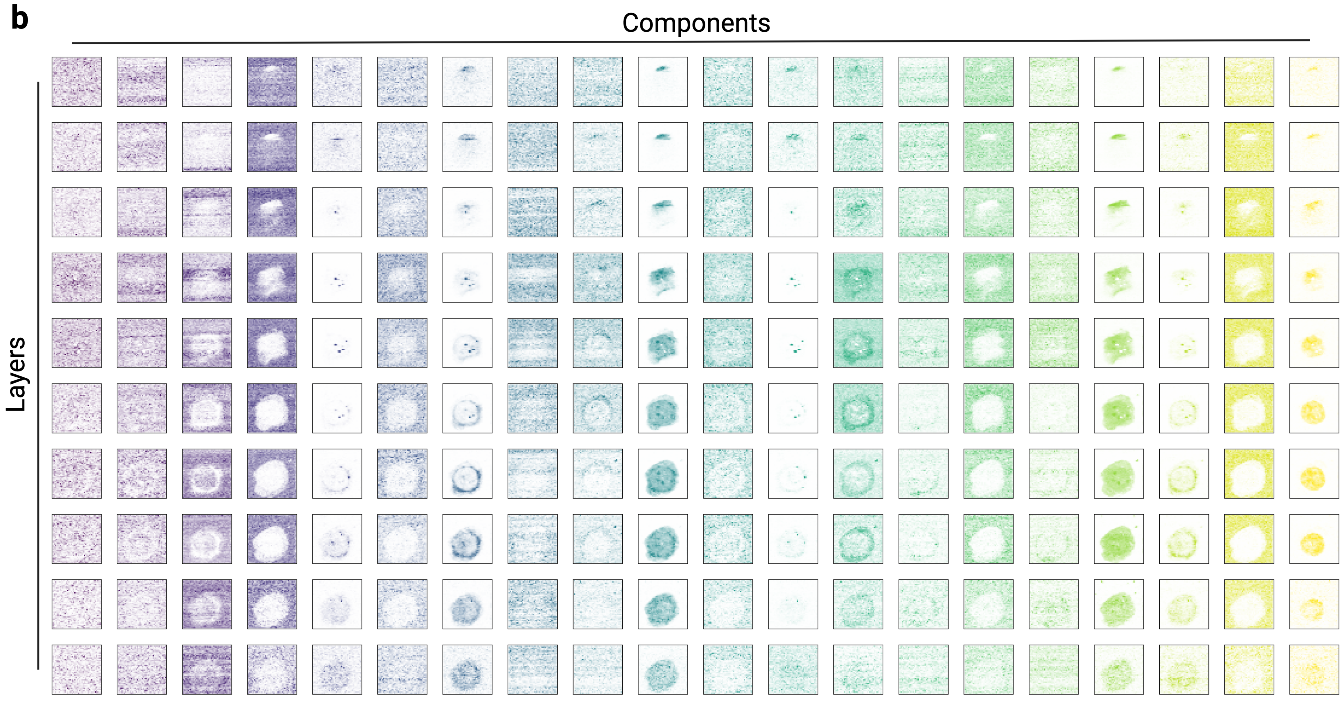


**Fig. S4. Full unmixing results obtained with our *Dense AE* model on the THP-1 cell data. a,** Derived endmembers. **b,** Derived fractional abundances.

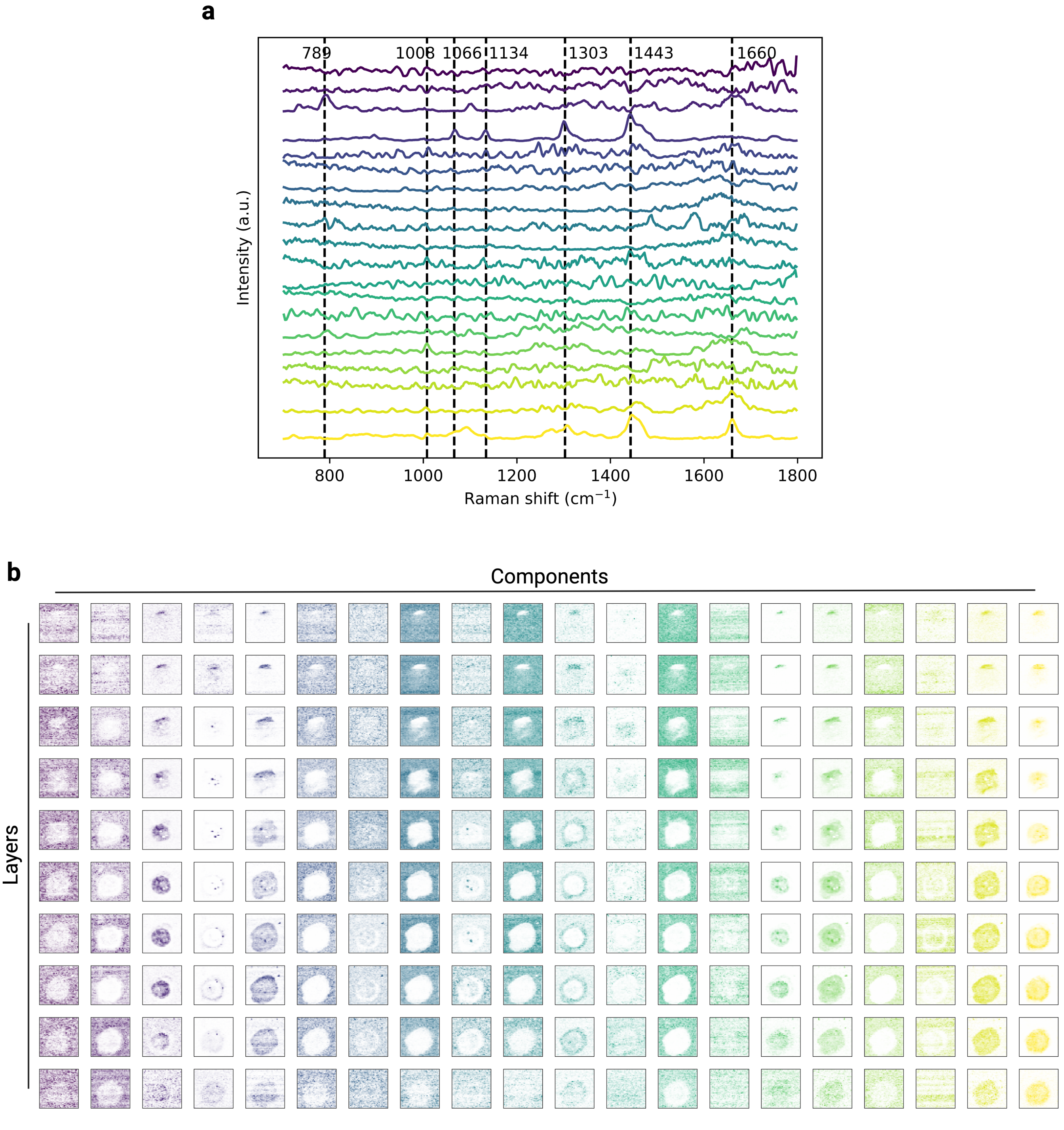


**Fig. S5. Full unmixing results obtained with our *Deep Dense AE* model on the preprocessed THP-1 cell data. a,** Derived endmembers. **b,** Derived fractional abundances.

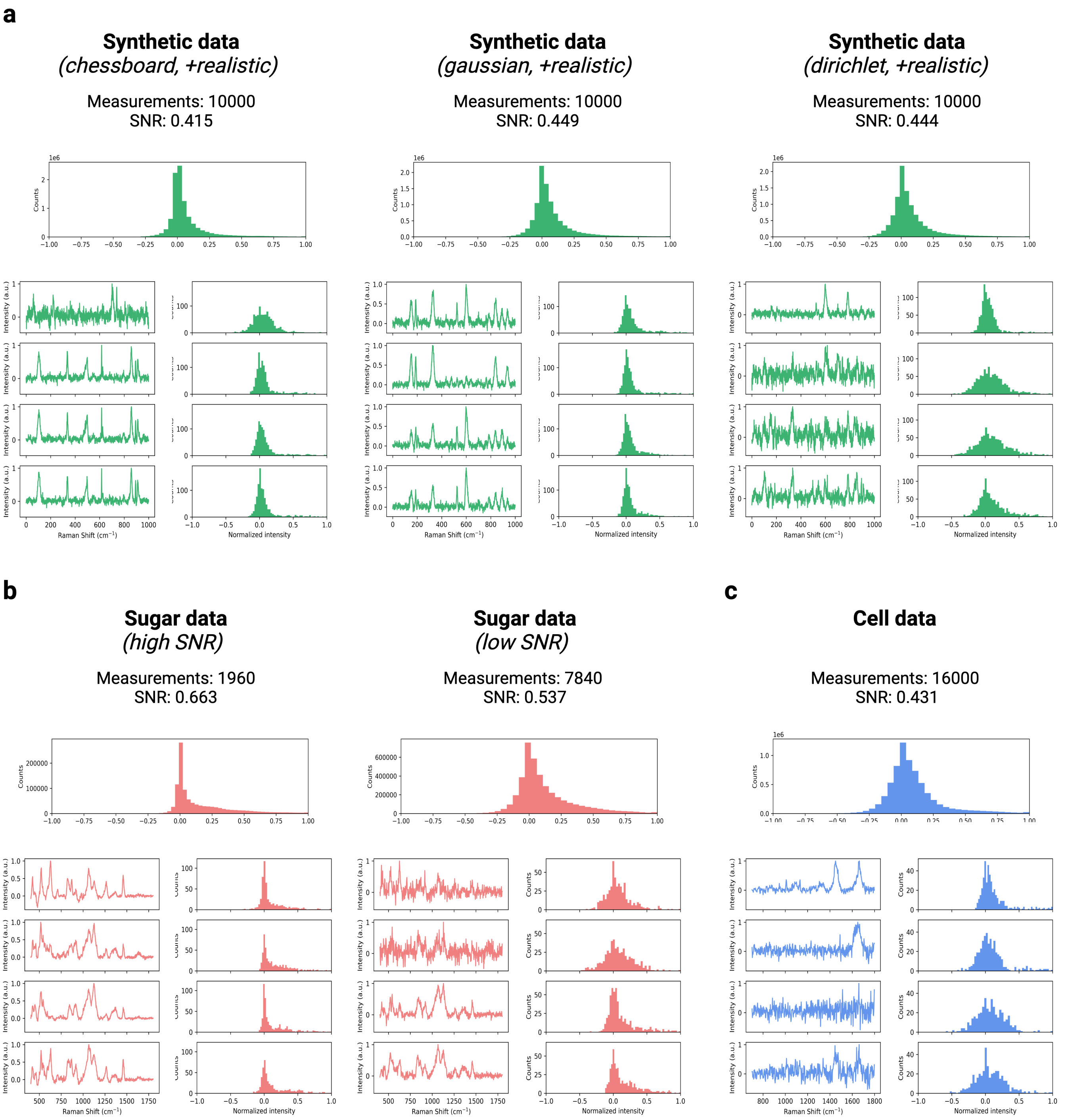


**Fig. S6. Key measures and statistics of the datasets used in this study.** Each panel provides a summary of key measures and statistics for a given dataset, including: the number of measurements present in the dataset and the mean signal-to-noise (SNR) ratio of the spectra; a histogram ($50$ bins) of all intensity values in the dataset; and $4$ randomly selected spectra from the dataset alongside their individual intensity histograms ($50$ bins). We provide summary information for three synthetic datasets (**a**), the two experimental datasets from sugar solutions with reference spectra (**b**), and the volumetric cell data (**c**). Before analysis, data were baseline corrected using Adaptive Smoothness Parameter Penalized Least Squares (see main text for parameters) and normalized by dividing each spectrum by its maximum value. Experimental data were also cropped to the fingerprint region $700–1800\,\mathrm{cm}^{-1}$ before preprocessing. The synthetic datasets (all *+realistic* scenarios) display comparable properties to real data.

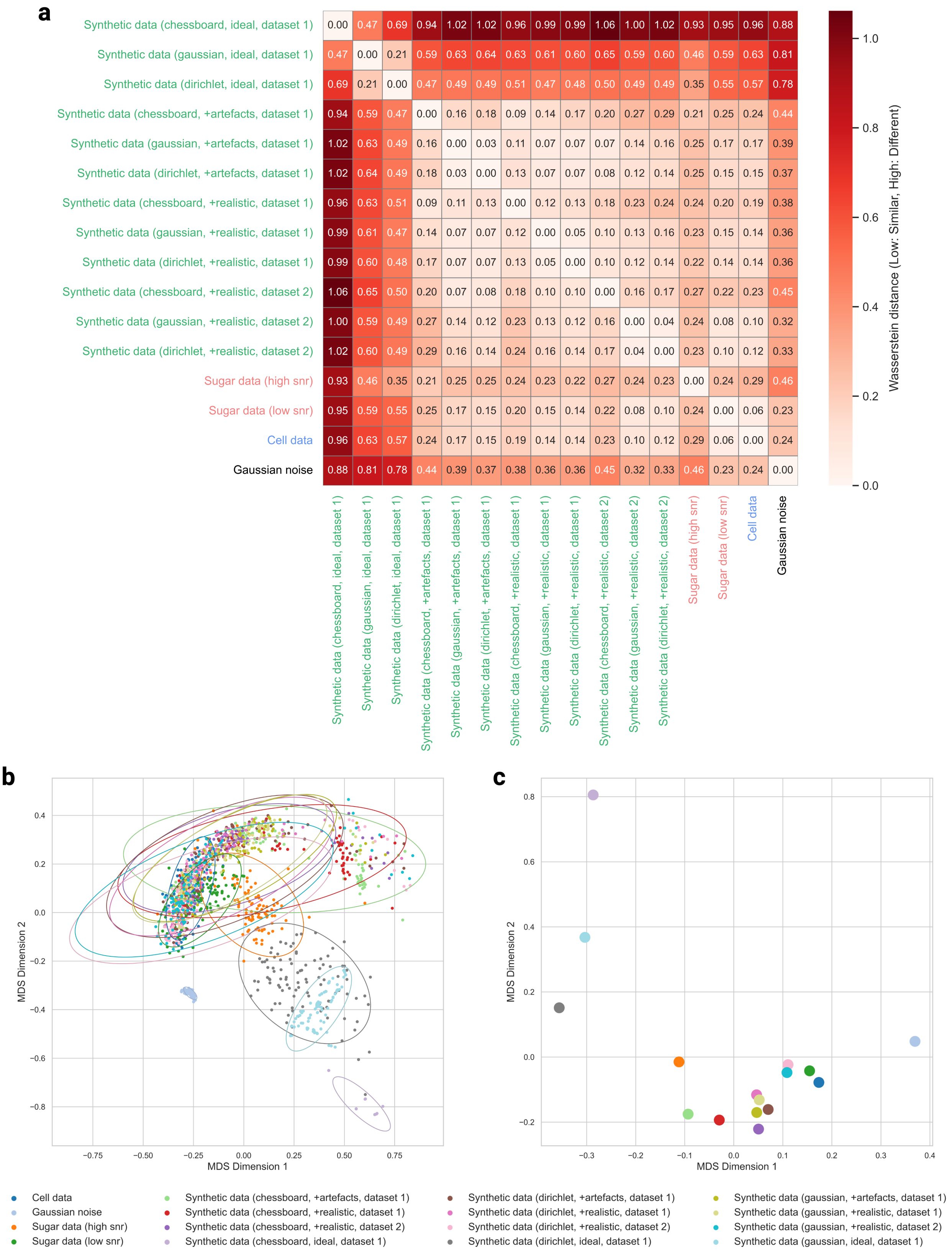


**Fig. S7. Similarity analysis of the spectral histograms of the datasets used in this study. a,** A matrix depicting the Wasserstein-1 distance between the dataset-wide intensity histograms ($50$ bins) of different synthetic and experimental datasets used in this study. 'Gaussian noise' represents a dataset that contains $10000$ 'spectra' each comprising $1000$ values independently sampled from a normal distribution with mean $0$ and standard deviation of $0.5$, added for reference. Synthetic data (*+artifacts* and *+realistic* scenarios) display low distances to real datasets. **b-c,** Multidimensional scaling (MDS) analysis of the calculated Wasserstein distances between the histograms of: $100$ randomly selected spectra from each considered dataset (**b**); and dataset-wide histograms (**c**). Ellipses in **b** represent 90% confidence regions for each group based on a multivariate Gaussian distribution model, showcasing the spread and overlap of the data points. The overlap of the ellipses in **b** indicates the high similarity of most of the synthetic datasets to the real data, as also shown by the proximity of the MDS projections of real data and synthetic data in **c**. Data preprocessing is described in Fig. S6.
